# Multicolor tunable emission through energy transfer in Dy^3+^/Ho^3+^ co-doped CaTiO_3_ phosphors with high thermal stability for solid state lighting applications

**DOI:** 10.1038/s41598-023-46065-4

**Published:** 2023-12-01

**Authors:** Priti Singh, Hirdyesh Mishra, Shyam Bahadur Rai

**Affiliations:** 1 Laser and Spectroscopy Laboratory, Department of Physics, Institute of Science, Banaras Hindu University, Varanasi, 221005 India; 2 Physics Section, Mahila Maha Vidhyalaya, Department of Physics, Banaras Hindu University, Varanasi, 221005 India

**Keywords:** Materials science, Optics and photonics

## Abstract

The exploration of multicolor emitting phosphors with single phase is extremely important for n-UV chip excited LED/WLED’s and multicolor display devices. In this paper, Dy^3+^, Ho^3+^ singly doped and Dy^3+^/Ho^3+^ co-doped CaTiO_3_ phosphor materials have been synthesized by solid state reaction method at 1473 K. The synthesized materials were characterized by XRD, FE-SEM, EDX, FTIR, PL and lifetime measurements. The PL emission spectra of Dy^3+^ doped CaTiO_3_ phosphors give intense blue and yellow emissions under UV excitation, while the PL emission spectra of Ho^3+^ doped CaTiO_3_ phosphor show intense green emission under UV/blue excitations. Further, to get the multicolor emission including white light, Dy^3+^ and Ho^3+^ were co-doped simultaneously in CaTiO_3_ host. It is found that alongwith colored and white light emissions, it also shows energy transfer from Dy^3+^ to Ho^3+^ with 367 nm and from Ho^3+^ to Dy^3+^ under 362 nm excitations. The energy transfer efficiency is found to be 67.76% and 69.39% for CaTiO_3_:4Dy^3+^/3Ho^3+^ and CaTiO_3_:3Ho^3+^/5Dy^3+^ phosphors, respectively. The CIE color coordinates, CCT and color purity of the phosphors have been calculated, which show color tunability from whitish to deep green via greenish yellow color. The lifetime of ^4^F_9/2_ level of Dy^3+^ ion and ^5^S_2_ level of Ho^3+^ ion is decreased in presence of Ho^3+^ and Dy^3+^ ions, respectively. This is due to energy transfer from Dy^3+^ to Ho^3+^ ions and vice versa. A temperature dependent photoluminescence studied of CaTiO_3_:4Dy^3+^/2Ho^3+^ phosphor show a high thermal stability (82% at 423 K of initial temperature 303 K) in the temperature range 303–483 K with activation energy 0.17 eV. The PLQY are 30%, 33% and 35% for CaTiO_3_:4Dy^3+^, CaTiO_3_:4Dy^3+^/2Ho^3+^ and CaTiO_3_:3Ho^3+^ phosphors, respectively. Hence, Dy^3+^, Ho^3+^ singly doped and Dy^3+^/Ho^3+^ co-doped CaTiO_3_ phosphor materials can be used in the field of single matrix perovskite color tunable phosphors which may be used in multicolor display devices, n-UV chip excited LED/WLED’s and photodynamic therapy for the cancer treatment.

## Introduction

Lanthanide ions doped phosphor materials produce multicolor tunable emissions under UV, visible or NIR excitations^1–3^, which have various applications in multicolor display devices, plasma displays panels (PDP) and light emitting diodes (LED) for solid-state lighting devices^4–6^. Lanthanides are also used in several other areas such as induced optical heating, optical thermometry, bio-imaging, lasers, solar cells and in photodynamic therapy to destroy cancer cells etc^7–11^.

The color tunable emission is very interesting property of lanthanide doped/co-doped phosphors. It can be observed by several ways, viz. by varying the concentration of dopants for same excitation wavelength or fixed dopants concentration and varying the excitation wavelength or through energy transfer from sensitizer to activator^12–14^. Chen et al. prepared Sm^3+^ doped Ca_2_NaZn_2_V_3_O_12_ phosphor by solid state reaction method and found the color tunabillity by tuning the Sm^3+^ ion concentration under 365 nm excitation^15^. Lohia et al. reported Eu^3+^ doped BaZnO_2_ phosphor and studied their photoluminescence emission under different excitation wavelengths (275, 370, 395 & 467 nm) in which the color was found to tune from whitish to red via orange one^16^. The multicolor tunable emission has been seen by many researchers via energy transfer from sensitizer to activator^17,18^. Förster’s and Dexter’s theories help to understand the energy transfer process from sensitizer (donor) to activator (acceptor) ions in organic and inorganic materials^19,20^. Qu et al. studied the color tunability in Tb^3+^ and Eu^3+^ co-doped Ca_2_YZr_2_Al_3_O_12_ phosphor via energy transfer from Tb^3+^ to Eu^3+^ ion^20^. They found that the color changes from green to yellow and then red by varying the concentration of Eu^3+^ ion. Li et al. have reported the color tunability from greenish yellow to red in KBaY(MoO_4_)_3_:Dy^3+^,Eu^3+^ phosphor through energy transfer from Dy^3+^ to Eu^3+^ ion^21^. Dwivedi et al. observed the multicolor tunable emission through energy transfer in Ho^3+^, Eu^3+^ co-doped Ca_0.05_Y_1.93-x_O_2_ nanophosphors^4^. Recently, Rai et al. have also observed color tunability from green to red via orange in LaVO_4_:Tb^3+^:Eu^3+^ phosphors on varying the concentration of Eu^3+^ ion^22^.

Among the lanthanide ions, Dy^3+^ and Ho^3+^ ions emit effective blue, yellow and green emissions, respectively in almost all hosts^3,6^. The emission from Dy^3+^ and Ho^3+^ ions individually under UV and blue excitations have been studied by several researchers in different hosts^23–27^. The blue emission of Dy^3+^ and green emission of Ho^3+^ may be used in photodynamic therapy for the cancer treatment^28–30^. The Dy^3+^ ions have been also used as an excellent sensitizer in different hosts^31,32^. Fu et al. successfully prepared the K_3_YB_6_O_12_:Dy^3+^, Eu^3+^ phosphor by solid state reaction method and have reported Dy^3+^ ion to behave as a sensitizer and transfer a part of its excitation energy to the Eu^3+^ ions^33^. Zhang et al. synthesized rare earth and transition metal combination (Ho^3+^/Bi^3+^) co-doped LaNbTiO_6_ phosphor via a facile sol–gel as well as combustion methods^34^. They found an energy transfer from Bi^3+^ to Ho^3+^ ions and blue to green color tunability. Recently, Guan et al. reported the Dy^3+^, Ho^3+^ co-doped NaGdF_4_ nanophosphor and studied energy transfer from Dy^3+^ to Ho^3+^ ion under 360 nm excitations^35^. They found color tunable emission in narrow region from blue to weak green with color coordinates to vary from (0.26, 0.33) to (0.27, 0.36). Therefore, we need to broad the color tunable region, which lie from whitish to deep green via greenish yellow. For this we choose Dy^3+^/Ho^3+^ co-doped CaTiO_3_ and studied the energy transfer, color tunability and temperature dependent photoluminescence in detail, which has not been studied till now to the best of our knowledge.

In the present work, we have prepared Dy^3+^, Ho^3+^ singly doped and Dy^3+^/Ho^3+^ co-doped CaTiO_3_ phosphors by solid state reaction method at 1473 K. The structural and morphological properties were studies by XRD, FE-SEM and EDX measurements. The vibrational bands due to different groups present in phosphor were analyzed by FTIR measurements. It is found that whereas Ho^3+^ emits intense green emission and weak red, Dy^3+^ gives intense blue and yellow emissions on UV excitation. We optimized the concentrations of Dy^3+^ and Ho^3+^ ions separately in this host to get maximum photoluminescence intensities. All the colors are emitted in Dy^3+^/Ho^3+^ co-doped CaTiO_3_ on UV excitations, which are color tunable with concentrations. It is also found that an energy transfer takes place from Dy^3+^ to Ho^3+^ on excitation with 367 nm in CaTiO_3_:4Dy^3+^/yHo^3+^ phosphors and from Ho^3+^ to Dy^3+^ on excitation with 362 nm in CaTiO_3_:3Ho^3+^/xDy^3+^ phosphors. The energy transfer efficiency and interaction involved have been studied in the two cases. The CIE coordinates calculations show that this phosphor emits white light and tunable greenish yellow to deep green color light with the variation in the concentration of Dy^3+^ and Ho^3+^ ions. The lifetime of ^4^F_9/2_ level of Dy^3+^ and ^5^S_2_ level of Ho^3+^ ions have been also measured in the absence and presence of Ho^3+^ and Dy^3+^ ions, respectively. The temperature dependent photoluminescence studies have been carried out to check the thermal stability behavior of the CaTiO_3_:4Dy^3+^/2Ho^3+^ phosphor. Thus, Dy^3+^, Ho^3+^ singly doped and Dy^3+^/Ho^3+^ co-doped CaTiO_3_ phosphor materials may be useful in achieving multicolor display devices, n-UV chip excited LED/WLED’s and in photodynamic therapy for the cancer treatments.

## Results and discussion

### Structural and morphological characterization

#### XRD measurements

**Figure 1:**
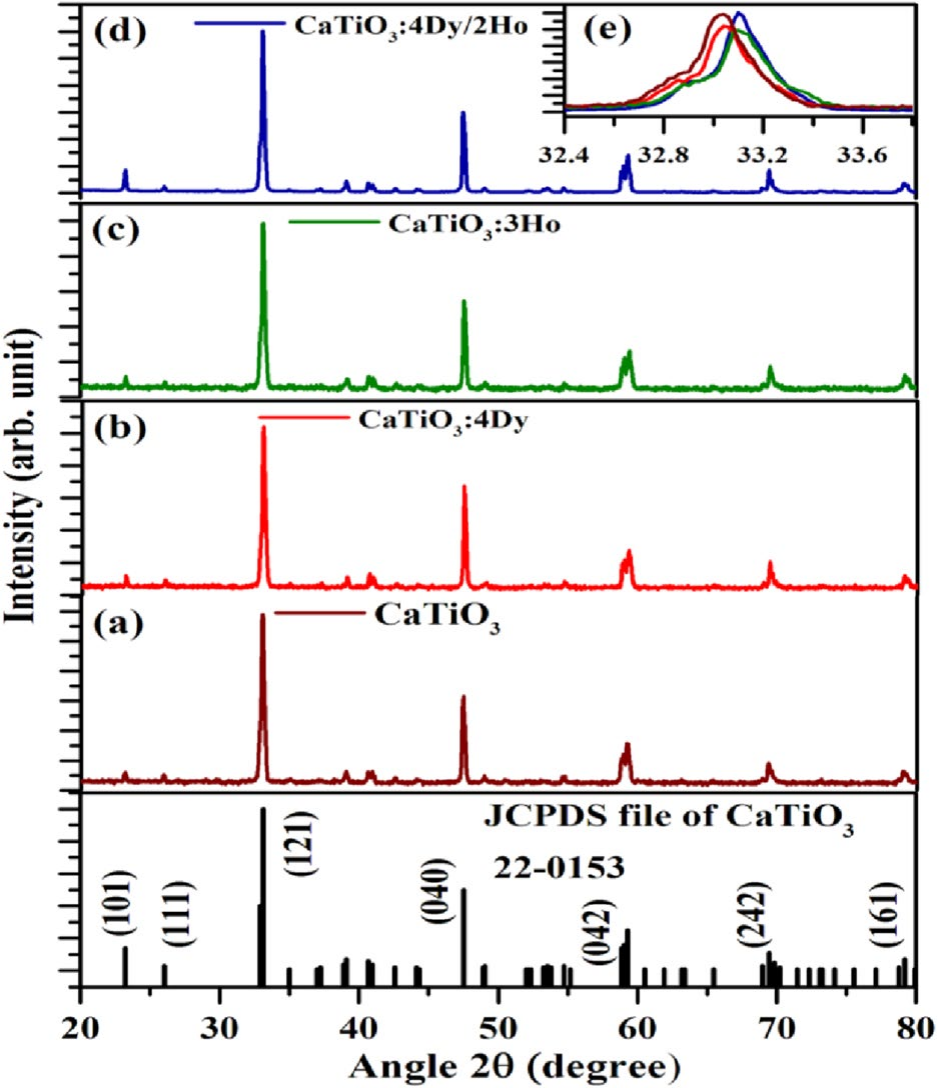
XRD patterns of (**a**) CaTiO_3_, (**b**) CaTiO_3_:4Dy^3+^, (**c**) CaTiO_3_:3Ho^3+^ and (**d**) CaTiO_3_: 4Dy^3+^/2Ho^3+^ phosphors (**e**) Zoomed XRD patterns in 32.4–33.8º range.

The X-ray diffraction patterns of the pure CaTiO_3_, CaTiO_3_:4Dy^3+^, CaTiO_3_:3Ho^3+^ and CaTiO_3_:4Dy^3+^/2Ho^3+^ phosphor samples were monitored in the 2θ range of 20-80º and it is shown in Fig. 1a–d along with its JCPDS file no—220153. The CaTiO_3_ host has orthorhombic phase and its space group is Pnma(62). All the doped phosphors show sharp diffraction peaks which are confirm highly crystalline nature of the phosphors. There is no additional diffraction peaks found by doping of 4Dy^3+^, 3Ho^3+^ and 4Dy^3+^/2Ho^3+^ in pure CaTiO_3_. Therefore, addition of 4Dy^3+^, 3Ho^3+^ and 4Dy^3+^/2Ho^3+^ ions in CaTiO_3_ donot affects the phase of the CaTiO_3_. Though doping of these ions at Ca^2+^ site, shift the diffraction peaks slightly towards higher 2θ angle side. This can be understood on the basis of their ionic radii. The ionic radii of Dy^3+^, Ho^3+^ and Ca^2+^ ions are 0.091, 0.090 and 0.100 nm, respectively. Therefore, on doping of Dy^3+^, Ho^3+^ ions at Ca^2+^ sites, crystal cell shrinks slightly due to which XRD peaks are shifted towards higher 2θ angle side. The effect of these shift for (121) peak is shown in Fig. 1e.

The value of average crystallite size (D) of pure CaTiO_3_, CaTiO_3_:4Dy^3+^, CaTiO_3_:3Ho^3+^ and CaTiO_3_:4Dy^3+^/2Ho^3+^ phosphors were calculated with the help of Debye–Scherrer (D–S) equation^36^:

1 $$\mathrm{D }= \frac{\mathrm{k \lambda }}{\mathrm{\beta cos\theta }},$$

where D is the crystallite size, k is the shape factor (0.89), λ is the X-ray wavelength, β is the full width at half maximum (FWHM) and θ is the diffraction angle. The average crystallite size values were found to be 30.0, 31.42, 32.12 and 31.70 nm for the pure CaTiO_3_, CaTiO_3_:4Dy^3+^, CaTiO_3_:3Ho^3+^ and CaTiO_3_:4Dy^3+^/2Ho^3+^ phosphors, respectively.

The micro-strain (e) has been also calculated for pure CaTiO_3_, CaTiO_3_:4Dy^3+^, CaTiO_3_:3Ho^3+^ and CaTiO_3_:4Dy^3+^/2Ho^3+^ phosphors by using the relation:

2 $$\mathrm{e }= \frac{\upbeta }{4\mathrm{ tan\theta }},$$

where the terms have their usual meaning. The micro-strain values for pure CaTiO_3_, CaTiO_3_:4Dy^3+^, CaTiO_3_:3Ho^3+^ and CaTiO_3_:4Dy^3+^/2Ho^3+^ phosphors were found to be 6.1 $\times$ 10^–5^, 5.6 $\times$ 10^–5^, 5.3 $\times$ 10^–5^ and 5.9 $\times$ 10^–5^, respectively.

#### FE-SEM and EDX measurements

**Figure 2:**
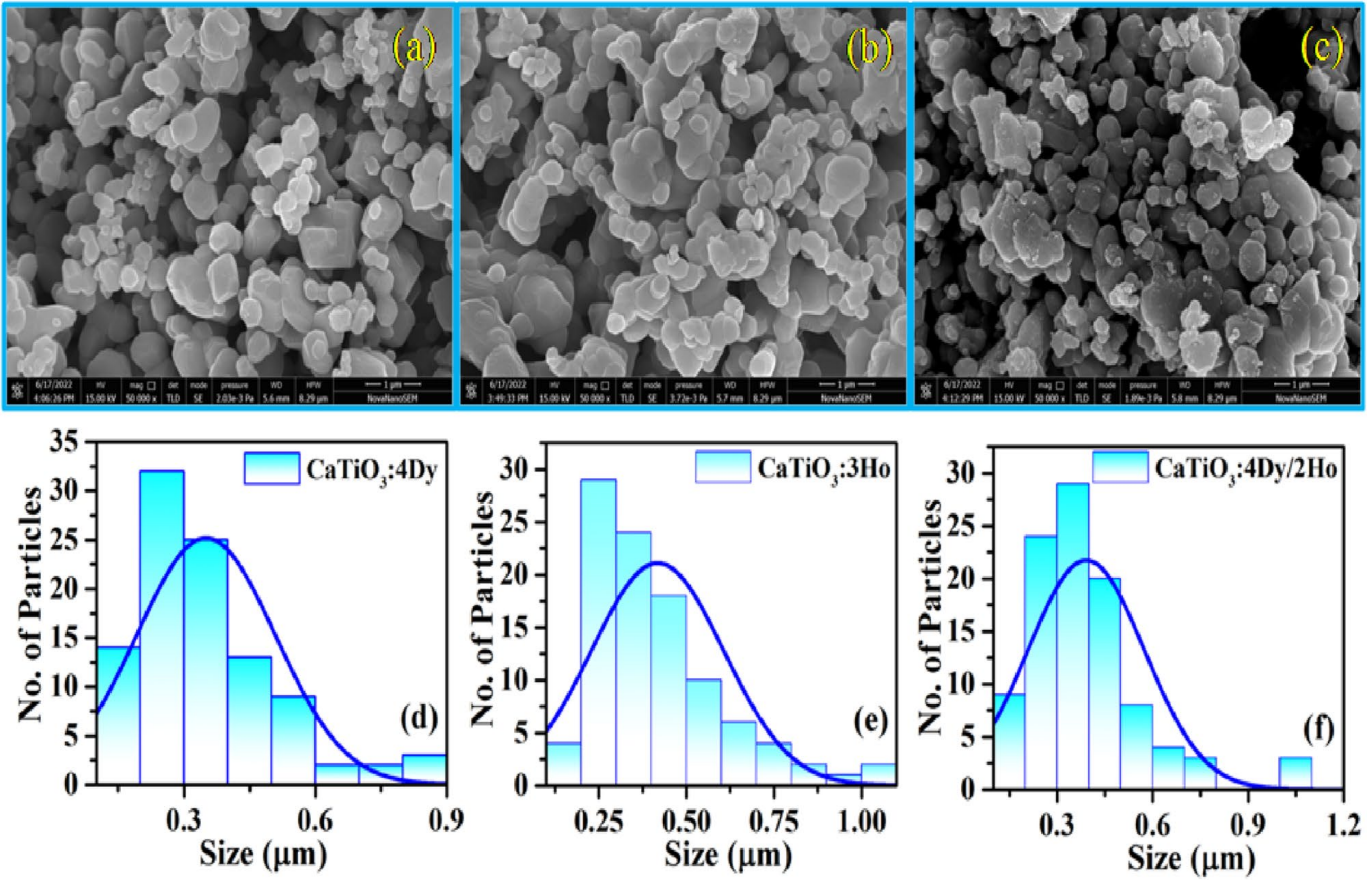
FE-SEM images of (**a**) CaTiO_3_:4Dy^3+^, (**b**) CaTiO_3_:3Ho^3+^, (**c**) CaTiO_3_:4Dy^3+^/2Ho^3+^ and histogram plots for particles size distribution of (**d**) CaTiO_3_:4Dy^3+^, (**e**) CaTiO_3_:3Ho^3+^ and (**f**) CaTiO_3_: 4Dy^3+^/2Ho^3+^ phosphors.

The surface morphology of CaTiO_3_:4Dy^3+^, CaTiO_3_:3Ho^3+^ and CaTiO_3_:4Dy^3+^/2Ho^3+^ phosphors are given in Fig. 2a–c, respectively. From the figure it is clear that particles are nearly spherical in shape and some particles are agglomerated with each other.

The average particles size of the CaTiO_3_:4Dy^3+^, CaTiO_3_:3Ho^3+^ and CaTiO_3_:4Dy^3+^/2Ho^3+^ phosphors were calculated by histogram plots using image j software [see Fig. 2d–f)] and the values obtained are 0.35, 0.41 and 0.39 µm, respectively. Chauhan et al. have calculated the average particles size by SEM images in which some particles are agglomerated. They have used image j software and the histogram plot method for calculate average particles size^37^. Our group have also used image j software and histogram plot method for calculate average particles size from the SEM images^38^.

**Figure 3:**
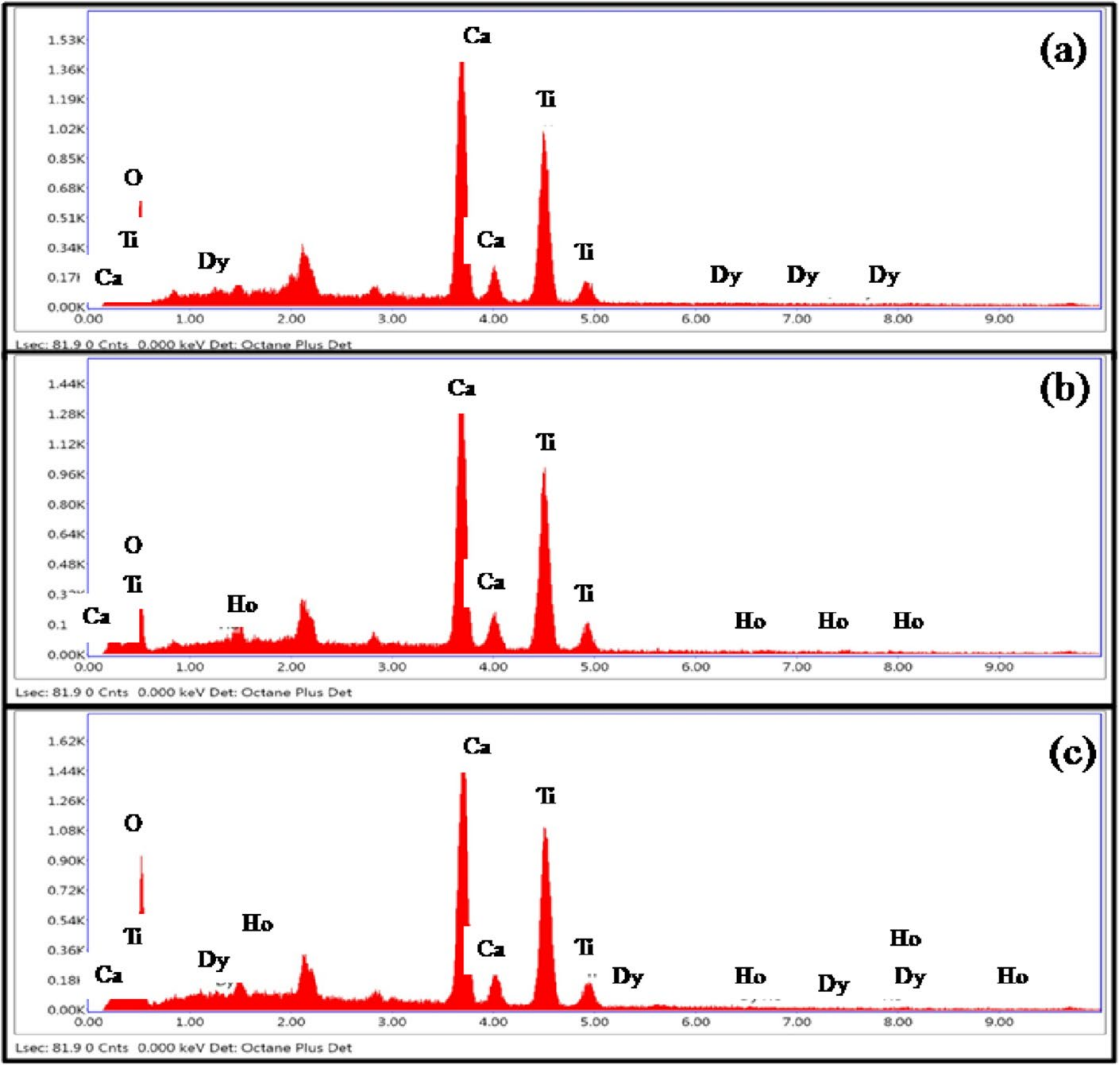
EDX spectra of (**a**) CaTiO_3_:4Dy^3+^, (**b**) CaTiO_3_:3Ho^3+^ and (**c**) CaTiO_3_: 4Dy^3+^/2Ho^3+^ phosphor samples.

The elements present in the prepared phosphors were verified by energy dispersive X-ray spectroscopic (EDX) measurements. Figure 3a–c shows the EDX patterns of CaTiO_3_:4Dy^3+^, CaTiO_3_:3Ho^3+^ and CaTiO_3_:4Dy^3+^/2Ho^3+^ phosphors, respectively. The EDX spectra show the presence of Ca, Ti, O, Dy and Ho elements in the phosphors. The Dy and Ho peaks are very weak appear in the EDX spectra due to its low concentrations. Several researchers also reported the EDX spectra in which the concentration of do-pants are low and due to this its peak appear very weak in EDX spectra^38–40^.

### Optical characterization

#### Fourier transform infrared spectra (FTIR)

**Figure 4:**
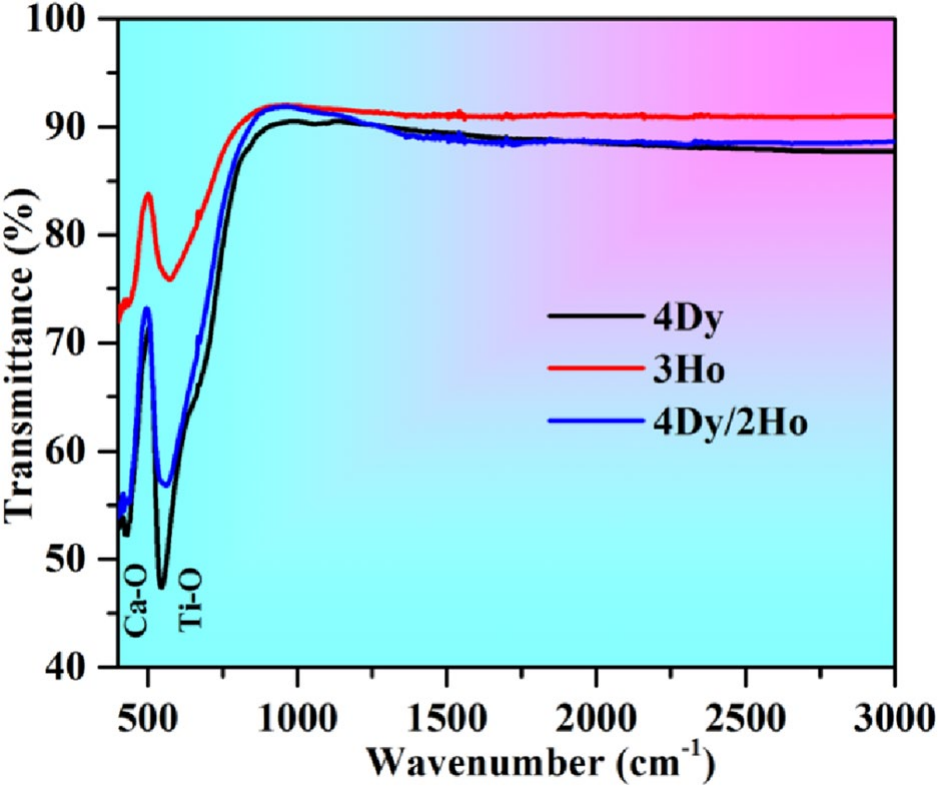
FTIR spectra of CaTiO_3_:4Dy^3+^, CaTiO_3_:3Ho^3+^ and CaTiO_3_:4Dy^3+^/2Ho^3+^ phosphor samples.

The FTIR spectra of the samples were used to categorize the vibrational bands due to different molecular groups present in the phosphor samples. The FTIR spectra of the different samples in the spectral range 400–3000 cm^−1^ are shown in the Fig. 4. The vibrational bands are observed at 433 and 541 cm^−1^, which corresponds to Ca-O and Ti–O groups^2,3^.

### Photoluminescence excitation and emission spectra of CaTiO_3_:xDy^3+^ phosphors

**Figure 5:**
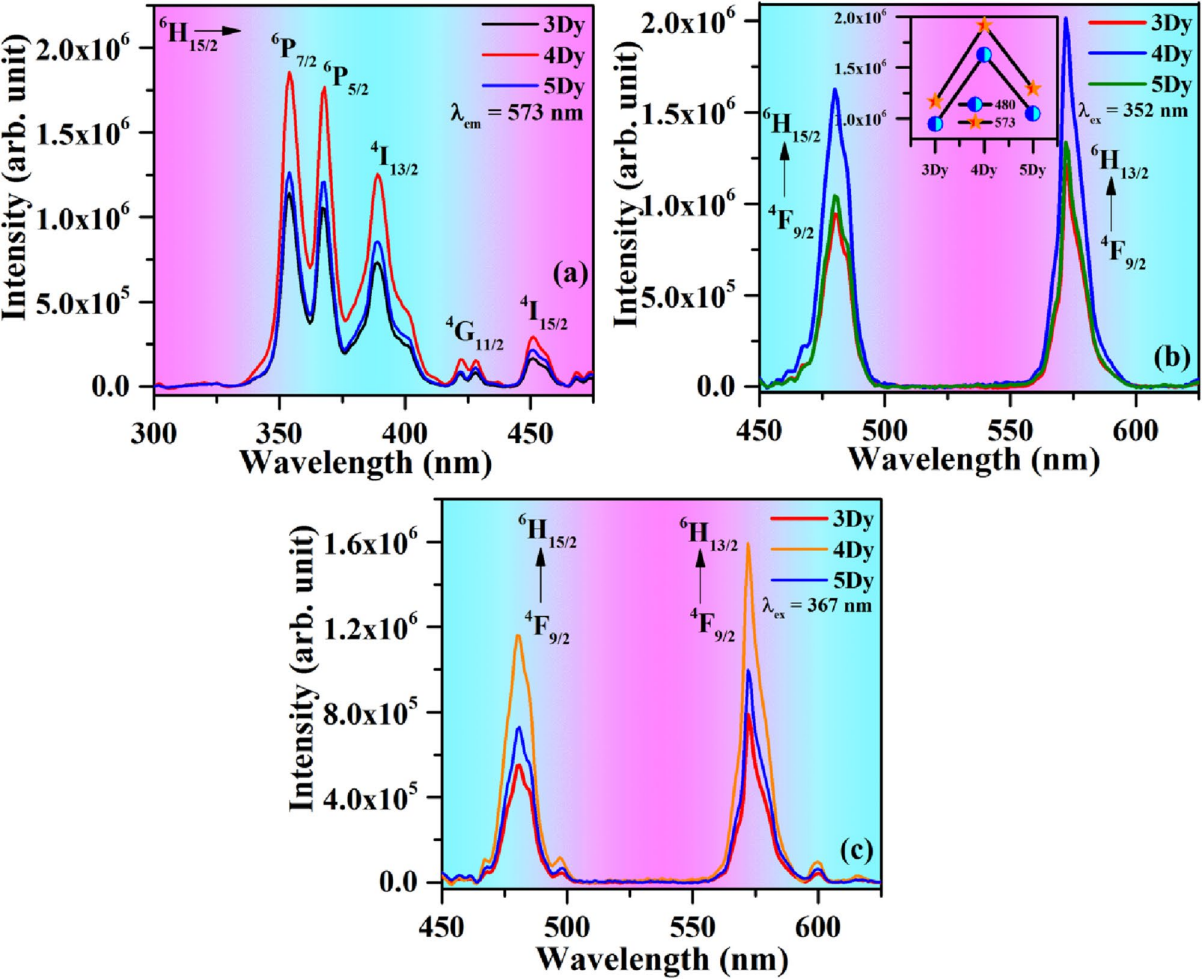
(**a**) Photoluminescence excitation (PLE) spectra of the xDy^3+^ doped CaTiO_3_ phosphors by monitoring at λ_em_ = 573 nm. PL emission spectra of the xDy^3+^ doped CaTiO_3_ phosphors with λ_ex_ = (**b**) 352 and (**c**) 367 nm.

The PL excitation spectra of CaTiO_3_:xDy^3+^ (where x = 3.0, 4.0 & 5.0 mol %) phosphor samples in the spectral region 300–475 nm with λ_em_ = 573 nm are shown in Fig. 5a. The PL excitation spectra show a number of peaks due to different transitions of Dy^3+^. The peaks of Dy^3+^ ions situated at 352, 367, 388, 430 and 460 nm, which could be assigned to arise due to ^6^H_15/2_ → ^6^P_7/2_, ^6^H_15/2_ → ^6^P_5/2_, ^6^H_15/2_ → ^4^I_13/2_, ^6^H_15/2_ → ^4^G_11/2_ and ^6^H_15/2_ → ^4^I_15/2_ transitions, respectively. The peaks at 352, 367 and 388 nm are intense compared to other peaks^1,41–43^. All these peaks wavelength lie in the emission range of n-UV LED chip. These peaks are therefore useful for LEDs applications.

The PL emission spectra of CaTiO_3_:xDy^3+^ phosphors on excitation with 352 and 367 nm wavelengths in the spectral region 450–625 nm are shown in Fig. 5b,c, respectively. The emission spectra contain two intense peaks at 480 and 573 nm due to ^4^F_9/2_ → ^6^H_15/2_ and ^4^F_9/2_ → ^6^H_13/2_ transitions, respectively^43–45^. The 573 nm peak is more intense than 480 nm peak. The intensity of emission is highest for 4 mol % concentration of Dy^3+^ ion (see inset in Fig. 5b). The intensity of the peaks decreases for higher concentrations due to concentration quenching. The emission intensity is larger for 352 nm excitation.

### Photoluminescence excitation and emission spectra of CaTiO_3_:yHo^3+^ phosphors

**Figure 6:**
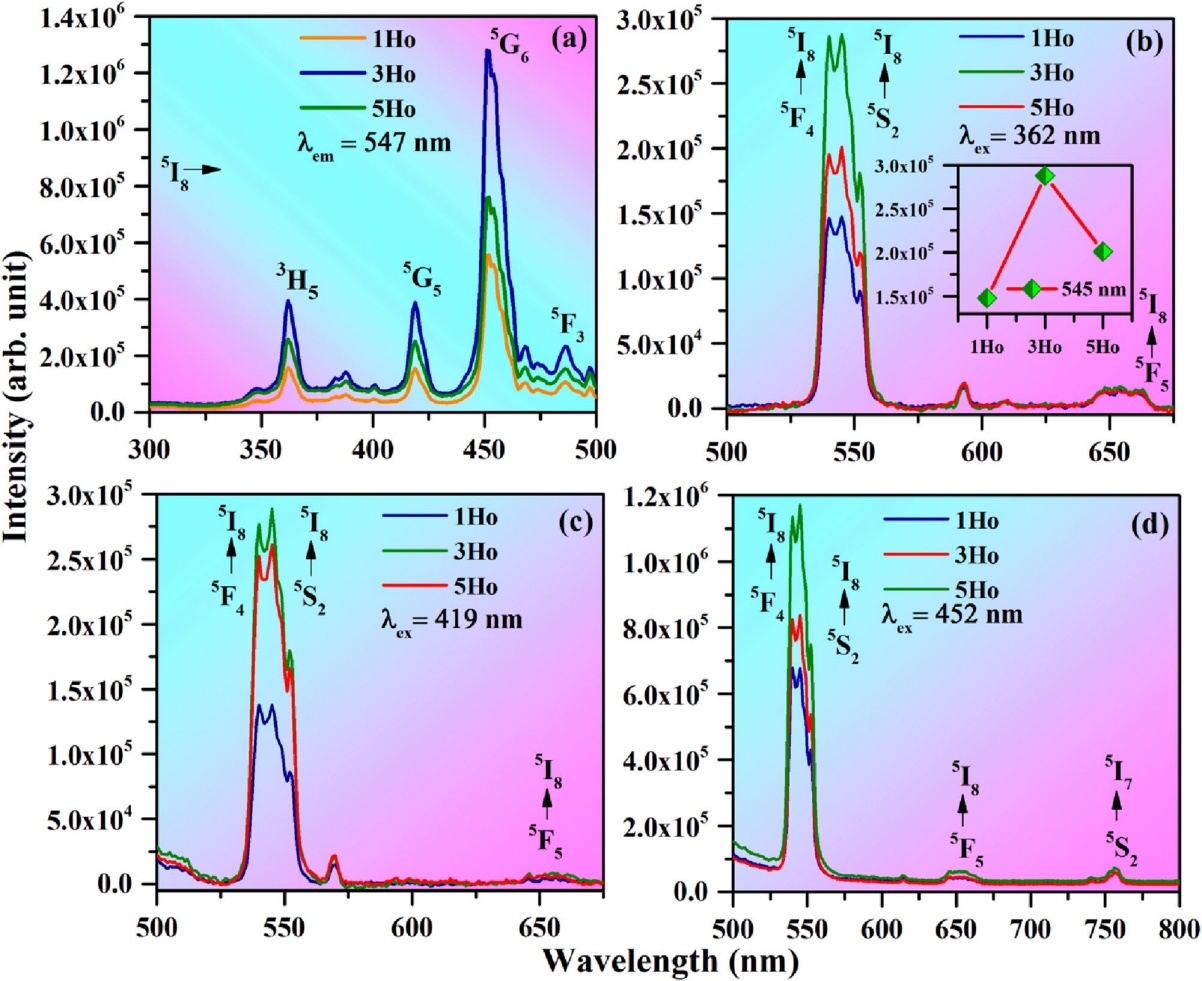
(**a**) Photoluminescence excitation (PLE) spectra of the yHo^3+^ doped CaTiO_3_ phosphors by monitoring λ_em_ = 547 nm. PL emission spectra of the yHo^3+^ doped CaTiO_3_ phosphors with (**b**) λ_ex_ = 362 nm, (**c**) λ_ex_ = 419 nm and (**d**) λ_ex_ = 452 nm.

Figure 6a shows the photoluminescence excitation (PLE) spectra of the yHo^3+^ (where y = 1.0, 3.0 & 5.0 mol %) doped CaTiO_3_ phosphors in the spectral region of 300–500 nm with λ_em_ fixed at 547 nm (corresponding to the ^5^S_2_ → ^5^I_8_ transition of Ho^3+^ ion). The spectra contain intense excitation peaks at 362, 419, 452 and 486 nm and they are assigned due to ^5^I_8_ → ^3^H_5,_^5^I_8_ → ^5^G_5_, ^5^I_8_ → ^5^G_6_ and ^5^I_8_ → ^3^F_3_ transitions of Ho^3+^ ions, respectively^3,46^. The excitation peak at 452 nm due to ^5^I_8_ → ^5^G_6_ transition appears with maximum intensity. The intensity of excitation peaks is optimum for 3 mol % of Ho^3+^ ions. Figure 6b–d show the PL emission spectra of the yHo^3+^ doped CaTiO_3_ phosphors on excitations with 362, 419 and 452 nm in the spectral region 500–675 and 500–800 nm, respectively. The PL emission spectra contain an intense green alongwith weak red and very weak NIR bands at 539/547, 652 and 756 nm corresponding to ^5^F_4_/^5^S_2_ → ^5^I_8_, ^5^F_5_ → ^5^I_8_ and ^5^S_2_ → ^5^I_7_ transitions of Ho^3+^ ions, respectively^3,46,47^. The intensity of green emission is dominated over the red and NIR emissions. The photoluminescence emission intensity is maximum at 3 mol % of Ho^3+^ ions concentration. For higher doping concentrations the emission intensity decrease due to concentration quenching effect (see inset of Fig. 6b). The PL emission intensity is largest for 452 nm excitation.

### Photoluminesence excitation and emission spectra of CaTiO_3_:4Dy^3+^/yHo^3+^ phosphors

**Figure 7:**
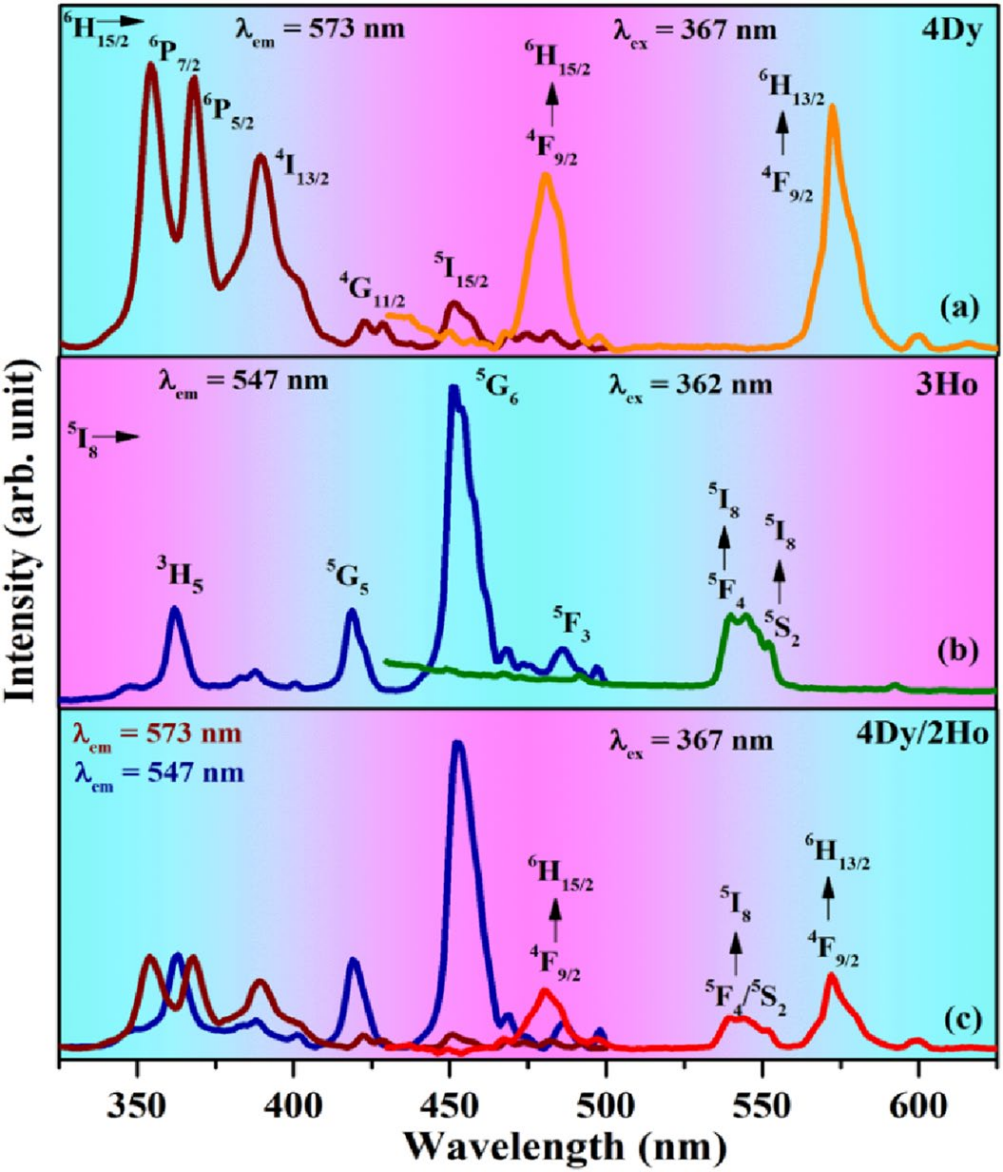
Photoluminescence excitation and emission spectra of (**a**) CaTiO_3_:4Dy^3+^, (**b**) CaTiO_3_:3Ho^3+^ and (**c**) CaTiO_3_:4Dy^3+^/2Ho^3+^ phosphors.

**Figure 8:**
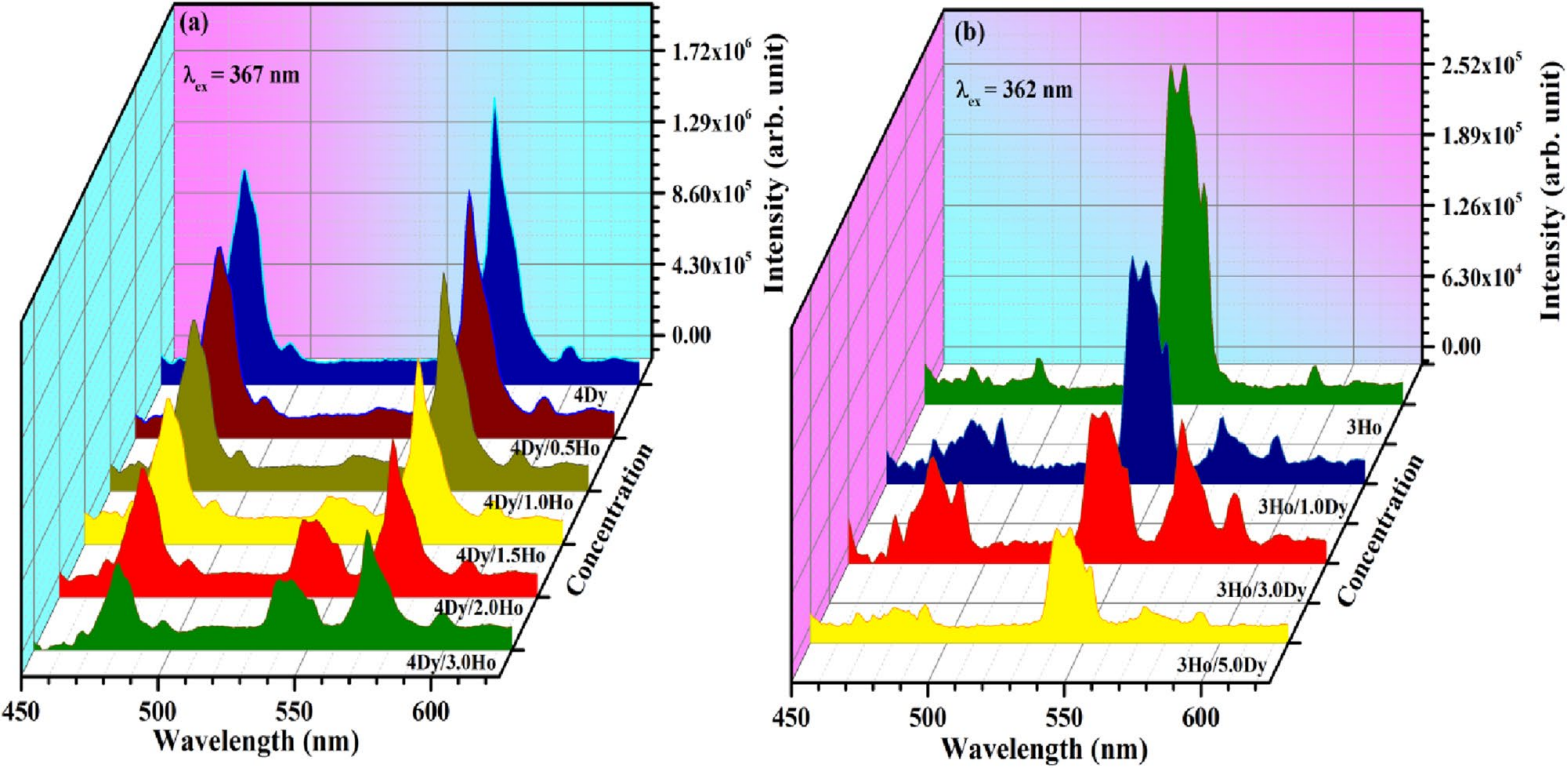
Photoluminescence emission (PL) spectra of (**a**) CaTiO_3_:4Dy^3+^/yHo^3+^ (where y = 0, 0.5, 1.0, 1.5, 2.0 & 3.0 mol %) phosphors with λ_ex_ = 367 nm and (**b**) CaTiO_3_:3Ho^3+^/xDy^3+^ (where x = 0, 1.0, 3.0 & 5.0 mol %) phosphors with λ_ex_ = 362 nm.

Photoluminescence excitation and emission spectra of CaTiO_3_:4Dy^3+^ and CaTiO_3_:3Ho^3+^ phosphors are shown in Fig. 7a,b**,** respectively. The transitions involved in the excitation and emission spectra are discussed earlier. Figure 7c shows the photoluminescence excitation and the emission spectra of CaTiO_3_:4Dy^3+^/2Ho^3+^ phosphor at λ_em_ = 547, 573 nm and λ_ex_ = 367 nm, respectively. The excitation peaks of Dy^3+^ and Ho^3+^ observed in mixed case (CaTiO_3_:4Dy^3+^/2Ho^3+^ phosphor) are exactly reproduced as in their individual cases. The 367 nm excitation in the mixed case gives blue and yellow as well as green emissions. This wavelength matches well with the level of Dy^3+^. The appearance of emission from Ho^3+^ ion under 367 nm is partially due to excitation of Ho^3+^ ion (because Ho^3+^ ion also weakly excited by 367 nm) and the rest due to the energy transfer from Dy^3+^ to Ho^3+^ ions. A similar type spectra are also seen on excitation with 362 nm (a wavelength which match exactly with Ho^3+^ level) where the Dy^3+^ emission also appears with the Ho^3+^ emission [see Fig. 8b]. This shows that color emitted by the phosphor sample can be tuned by appropriate doping ratio of Dy^3+^/Ho^3+^ ions in CaTiO_3_ and selecting proper excitation wavelength.

The energy transfer from sensitizer to activator ions can occur when the excitation spectrum of the activator and emission spectrum of the sensitizer overlap on each other^48^. Figure 7a,b clearly shows that the exication peak due to ^5^I_8_ → ^5^F_3_ transition (486 nm) of Ho^3+^ ions overlap with the emission peak due to ^4^F_9/2_ → ^6^H_15/2_ transition (480 nm) of Dy^3+^ ions. This clearly indicates that an energy transfer is possible from Dy^3+^ to Ho^3+^ ions.

In order to understand the effect of Ho^3+^ ion concentrations on the photoluminescence intensity of Dy^3+^, energy transfer efficiency and color tunability, we synthesized the CaTiO_3_:4Dy^3+^/yHo^3+^ (where y = 0, 0.5, 1.0, 1.5, 2.0 & 3.0 mol%) phosphors, and monitored their photoluminescence emission spectra with λ_ex_ = 367 nm. The spectra obtained are shown in Fig. 8a. It can be seen that the PL emission intensity of Dy^3+^ ions decreased while that of Ho^3+^ ions increases with increasing the concentrations of Ho^3+^ ions and this is due to energy transfer from Dy^3+^ to Ho^3+^ ions. A reverse energy transfer (i.e. from Ho^3+^ to Dy^3+^ ions) is also possible for 362 nm excitation (due to overlapping of 362 nm band of Ho^3+^ and 367 nm band of Dy^3+^ [see Fig. 7c)]. For this we co-doped xDy^3+^ (where x = 0, 1.0, 3.0 & 5.0 mol %) in CaTiO_3_:3Ho^3+^ phosphor and this is shown in Fig. 8b. The emission intensity of Ho^3+^ ions decrease with the increase of the concentration of Dy^3+^ ions. This clearly shows the energy transfer from Ho^3+^ to Dy^3+^ ions.

**Figure 9:**
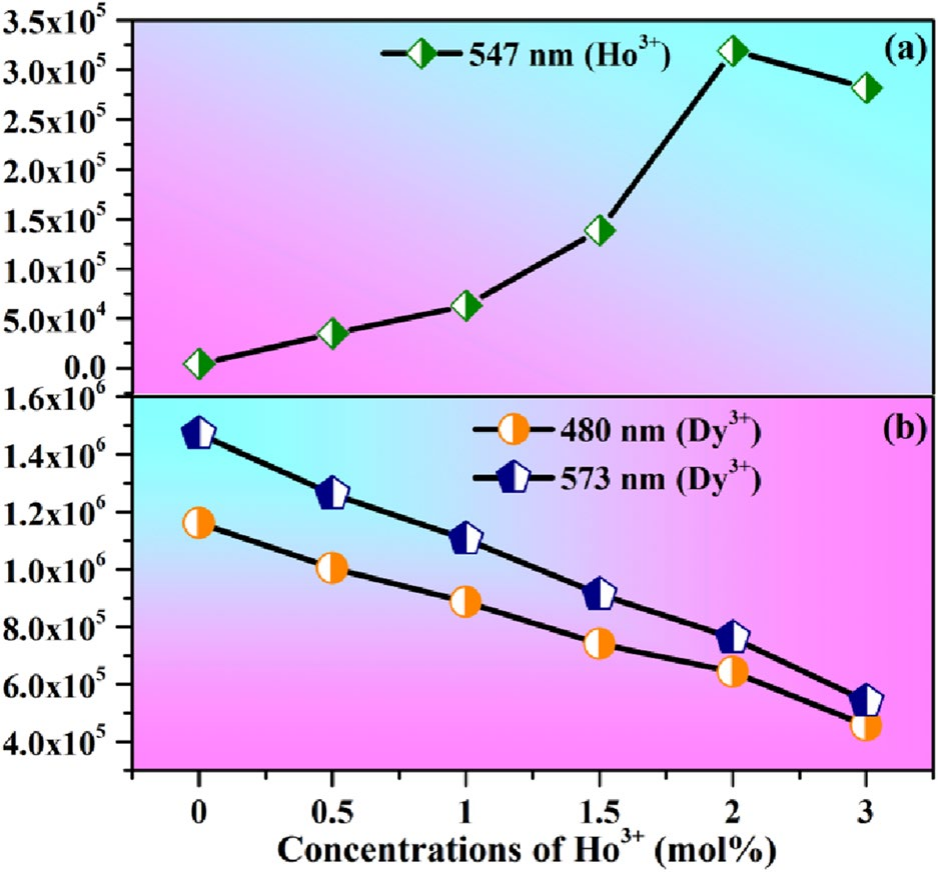
(**a**,**b**) Variation in emission intensities of Ho^3+^ and Dy^3+^ ions bands with the variation of concentrations of Ho^3+^ ion in CaTiO_3_:4Dy^3+^/yHo^3+^ phosphors under 367 nm excitation.

To understand the variation in emission intensity, the emission intensity of Ho^3+^ and Dy^3+^ ions with different concentrations of Ho^3+^ ions and fixed concentration of Dy^3+^ [i.e. 4Dy^3+^/yHo^3+^ (where y = 0, 0.5, 1.0, 1.5, 2.0 & 3.0 mol%) co-doped CaTiO_3_ phosphors] are shown in Fig. 9a,b). From the figure it is obvious that the emission intensity of Ho^3+^ ions increase while that of Dy^3+^ ion decrease with increasing the concentration of Ho^3+^ ions in 4Dy^3+^/yHo^3+^ co-doped CaTiO_3_ phosphors and it is maximum for 2 mol% of Ho^3+^ ions.

### Energy transfer efficiency from Dy^3+^ to Ho^3+^ and Ho^3+^ to Dy^3+^ ions and nature of interaction

**Figure 10:**
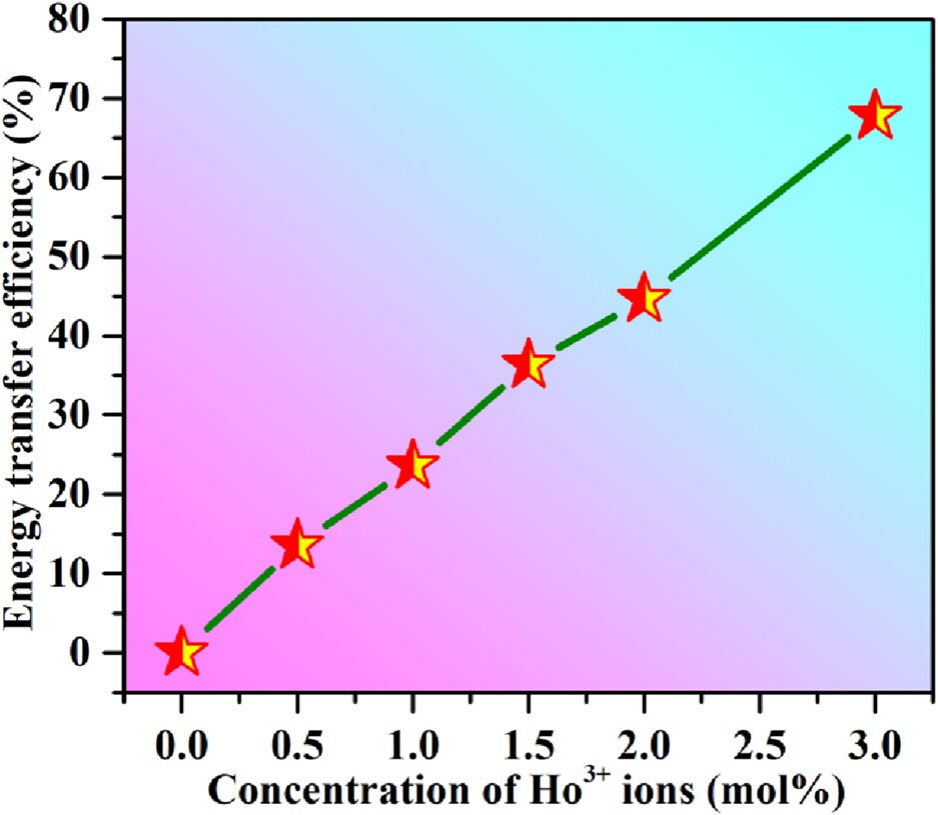
Energy transfer efficiency from Dy^3+^ to Ho^3+^ ion in CaTiO_3_:4Dy^3+^/yHo^3+^ (where y = 0.0, 0.5, 1.0, 1.5, 2.0 & 3.0 mol %) phosphors.

The energy transfer efficiency from Dy^3+^ to Ho^3+^ and Ho^3+^ to Dy^3+^ ions under 367 nm and 362 nm excitations can be calculated using the equation^4^:

3 $${\eta }_{T}=\left(1-\frac{I}{{I}_{0}}\right)\times 100\%,$$

where I and I_0_ are the emission intensities of sensitizer in the presence and absence of activator ion, respectively. Here, ${\eta }_{T}$ represents the energy transfer efficiency. Under the 367 nm excitation the energy transfer efficiency with different Ho^3+^ ion concentrations is shown in Fig. 10. The value of energy transfer efficiency is 0, 13.55, 23.66, 36.32, 44.6 and 67.76% for CaTiO_3_:4Dy^3+^/yHo^3+^ (where y = 0, 0.5, 1.0, 1.5, 2.0 & 3.0 mol%) phosphors.

The energy transfer efficiency has been also calculated in the case of CaTiO_3_:3Ho^3+^/xDy^3+^ (where x = 0, 1.0, 3.0 & 5.0 mol%) phosphors under 362 nm excitation and the values are 0, 36.42, 59.56 and 69.39%, respectively.

Generally, the energy transfer from sensitizer (donor) to activator (acceptor) ions can be studied by the Förster’s (FRET)/Dexter’s theories in organic and inorganic materials^19,20^. These studied have many pros and cons in the advanced material design for different applications. The FRET usually observed in organic materials design and use in cell biology, medicine, WLED etc^19,49,50^. The energy transfer efficiency in FRET depends upon refractive index of solvent, dipole orientation and quantum yield of the donor etc^19^. However, Dexter’s theory usually used to understand the non-radiative energy transfer due to exchange or multipolar interaction in oxide phosphors^21^. If the value of critical distance less than 5 Å, it is exchange interaction. However, if the value of critical distance is greater than 5 Å, it is multipolar interaction. In order, to understand the energy transfer interaction from sensitizer (Dy^3+^) to activator (Ho^3+^) ions in the CaTiO_3_:4Dy^3+^/yHo^3+^ phosphors, the value of critical distance needs to be evaluated. The value of critical distance between Dy^3+^-Ho^3+^ ions could be calculated by using the relation^51^:

4 $${\mathrm{R}}_{\mathrm{c}} = 2\left[\frac{3V}{4\pi \mathrm{x}N}\right]1/3,$$

where V is the volume of the unit cell, N is the number of Ca site. The critical concentration x is defined as the total concentration of Dy^3+^ and Ho^3+^ ions, when the emission intensity of Dy^3+^ with Ho^3+^ is half of that without Ho^3+^ doping. Here in, x is approximately ~ 0.063, V = 224.0342 Å^3^ and N = 4. The calculated value of R_c_ is found to be 11.44 Å. This value is greater than 5 Å which indicates that the energy transfers from sensitizer (Dy^3+^) to activator (Ho^3+^) ion is due to multipolar interaction. Dwivedi et al. have also studied the energy transfer from Ho^3+^ to Eu^3+^ in Ca_0.05_Y_1.93−x_O_2_ host. They have reported the value of critical distance is 15.14 Å and have calculated the multipolar interaction by Dexter’s formula and Reisfield’s approximation^4^.

**Figure 11:**
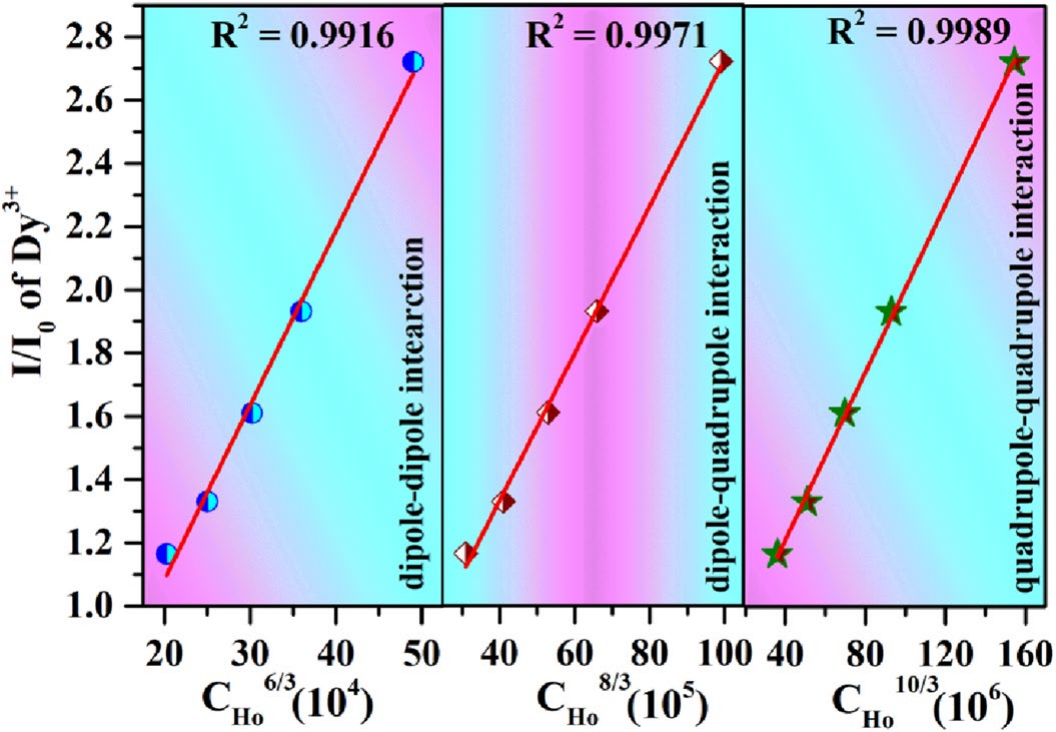
Plots of I/I_0_ vs C^n/3^ (where n = 6, 8 & 10) for CaTiO_3_:4Dy^3+^/yHo^3+^ phosphors.

The multipolar interaction can be determined by Dexter’s formula for energy transfer and Reisfield’s approximation^52^:

5 $$\frac{I}{{I}_{0}}= {\mathrm{C}}^{\mathrm{n}/3},$$

where I and I_0_ are the emission intensities of Dy^3+^ peak in the absence and presence of Ho^3+^ ions. C is the critical concentrations of Dy^3+^ and Ho^3+^ ions and n = 6, 8 and 10 for dipole–dipole (d–d), dipole–quadrupole (d–q) and quadrupole–quadrupole (q–q) interactions, respectively. The plot of I/I_0_ versus concentration are given in Fig. 11. A better fitting is observed for n = 10. Hence, the energy transfers from Dy^3+^ to Ho^3+^ ions is due to the quadrupole–quadrupole (q-q) interactions. This interaction in the case of energy transfer from Ho^3+^ to Dy^3+^ is found to be due to dipole–dipole.

### Energy level diagram of Dy^3+^, Ho^3+^ ions and the mechanism of energy transfer from Dy^3+^ to Ho^3+^ ions/Ho^3+^ to Dy^3+^ ions

**Figure 12:**
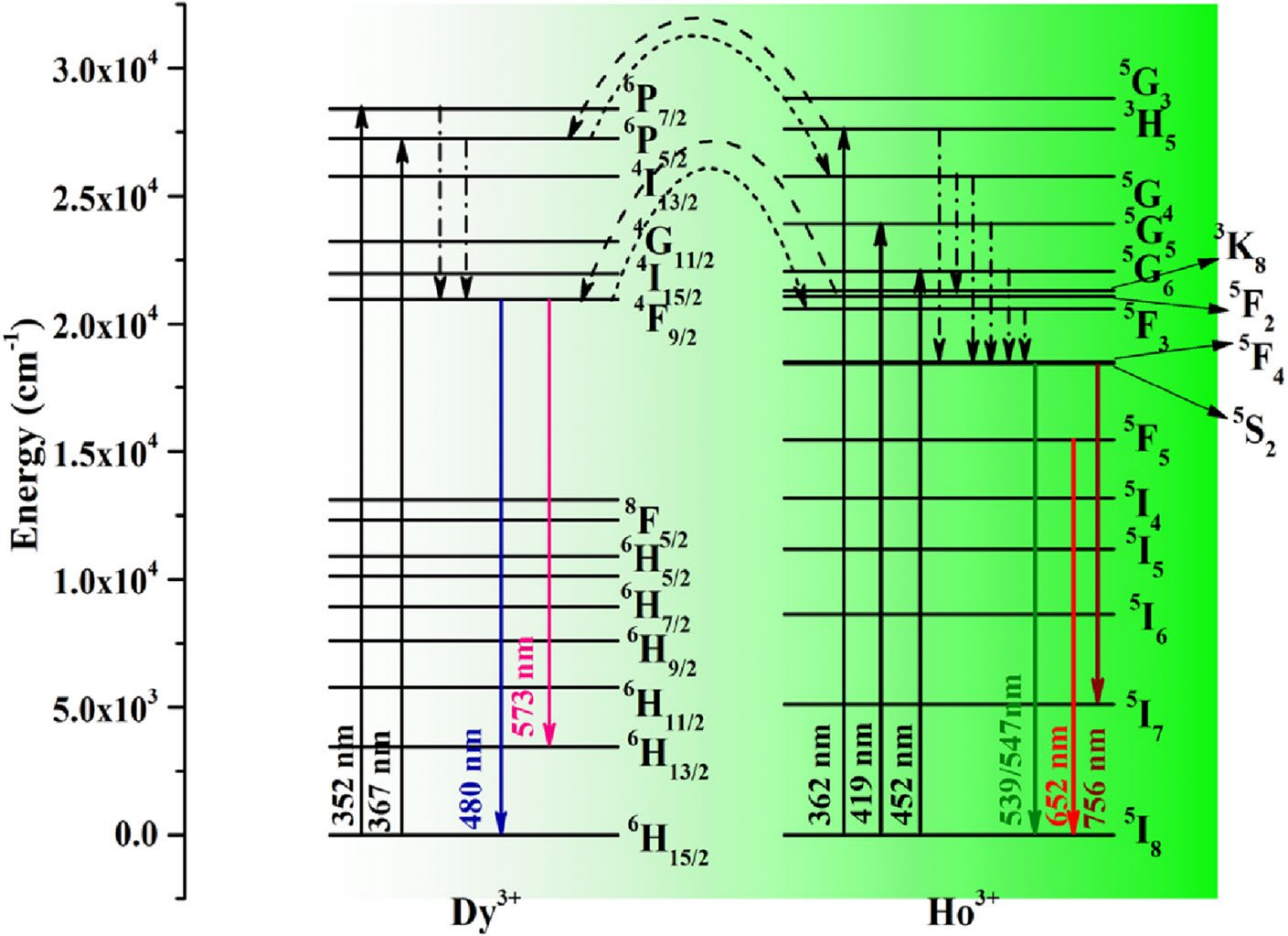
Schematic energy level diagrams of Dy^3+^, Ho^3+^ ions and energy transfer mechanism involved from Dy^3+^ to Ho^3+^ and Ho^3+^ to Dy^3+^ ions in photoluminescence process.

The excitation and emission spectra of Dy^3+^ and Ho^3+^ ions in Figs. 5 and 6 can be understood easily on the basis of energy level diagram shown in Fig. 12. The Dy^3+^ ions present in the ground state (^6^H_15/2_), on excitation with 352 and 367 nm it promoted to the ^6^P_7/2_ and ^6^P_5/2_ excited states, respectively. The excited Dy^3+^ ions from ^6^P_7/2_ and ^6^P_5/2_ state populate the lowest excited ^4^F_9/2_ state through several non-radiative relaxation processes. From ^4^F_9/2_ state, Dy^3+^ ions give emissions at 480 (blue) and 573 nm (yellow) by ^4^F_9/2_ → ^6^H_15/2_ and ^4^F_9/2_ → ^6^H_13/2_ transitions, respectively.

Similarly, the Ho^3+^ ions in ground state (^5^I_8_), on excitation with 362, 419 and 452 nm are promoted to the ^3^H_5,_^5^G_5_ and ^5^G_6_ excited states, respectively. The excited Ho^3+^ ions from these states relax to the ^5^F_4_/^5^S_2_ and ^5^F_5_ states. The Ho^3+^ ions from ^5^F_4_/^5^S_2_ states give strong green emissions at 539 and 547 nm through ^5^F_4_/^5^S_2_ → ^5^I_8_ transitions. The ions in ^5^S_2_ state also give emission in NIR region at 756 nm due to ^5^S_2_ → ^5^I_7_ transition. The ^5^F_5_ state also populated through non-radiative relaxation from ^5^F_4_/^5^S_2_ states gives relatively a weak red emission at 652 nm due to ^5^F_5_ → ^5^I_8_ transition of Ho^3+^ ion.

However, when Dy^3+^ and Ho^3+^ both ions are present in the sample simultaneously, an energy transfer takes place from Dy^3+^ to Ho^3+^ ions on 367 nm excitation due to which emission intensity of Ho^3+^ bands increased and that of Dy^3+^ decreased. The energy transfer mechanism from Dy^3+^ to Ho^3+^ ions can be understood though the energy level diagram shown in the Fig. 12. Actually when Ho^3+^ ions are co-doped in CaTiO_3_:Dy^3+^ phosphor, a part of energy is transferred from ^6^P_5/2_ state of Dy^3+^ to ^5^G_4_ state of Ho^3+^ ions under 367 nm excitations. The Ho^3+^ ions from ^5^G_4_ excited state relax non radiatively to the ^5^F_4_/^5^S_2_ excited states and give green emission at 539/547 nm due to ^5^F_4_/^5^S_2_ → ^5^I_8_ transitions. Another possibility of energy transfers from Dy^3+^ to Ho^3+^ may be that the excited Dy^3+^ ions from ^6^P_5/2_ state relax non radiatively to ^4^F_9/2_ (lowest excited state) and transfer a part of its excitation energy from this state to the ^5^F_3_ state of Ho^3+^ ion and loose another part radiatively to give radiative transitions ^4^F_9/2_ → ^6^H_15/2_ (blue) and ^4^F_9/2_ → ^6^H_13/2_ (yellow) with smaller intensity. The excited Ho^3+^ ions in ^5^F_3_ state relax non-radiatively to the ^5^F_4_/^5^S_2_ states which finally gives green emission.

As we have mentioned earlier, on excitation with 362 nm, a reverse energy transfer (i.e. from Ho^3+^ to Dy^3+^ ions) occurs and the mechanism involved can be understood again on the basis of energy level diagram shown in Fig. 12. Ho^3+^ ions transfer a part of their excitation energy from ^3^H_5_ state (Ho^3+^) to ^6^P_5/2_ state (Dy^3+^) under 362 nm excitations in CaTiO_3_:3Ho^3+^/xDy^3+^ phosphors. The Dy^3+^ ions from this state relax non-radiatively to the ^4^F_9/2_ excited state and give blue and yellow emissions. Another possibility of energy transfer from Ho^3+^ to Dy^3+^ is that the excited Ho^3+^ ions from ^3^H_5_ state relax non radiatively to ^5^F_2_ state and from there Ho^3+^ ions transfer a part of their excitation energy to the ^4^F_9/2_ state of Dy^3+^ ion. Thus, on excitation with 367/362 nm, we get bands due to Dy^3+^ as well as Ho^3+^ both ions via energy transfer from Dy^3+^ to Ho^3+^ ions and vice versa.

### Color coordinates, CCT and color purity calculations

**Figure 13:**
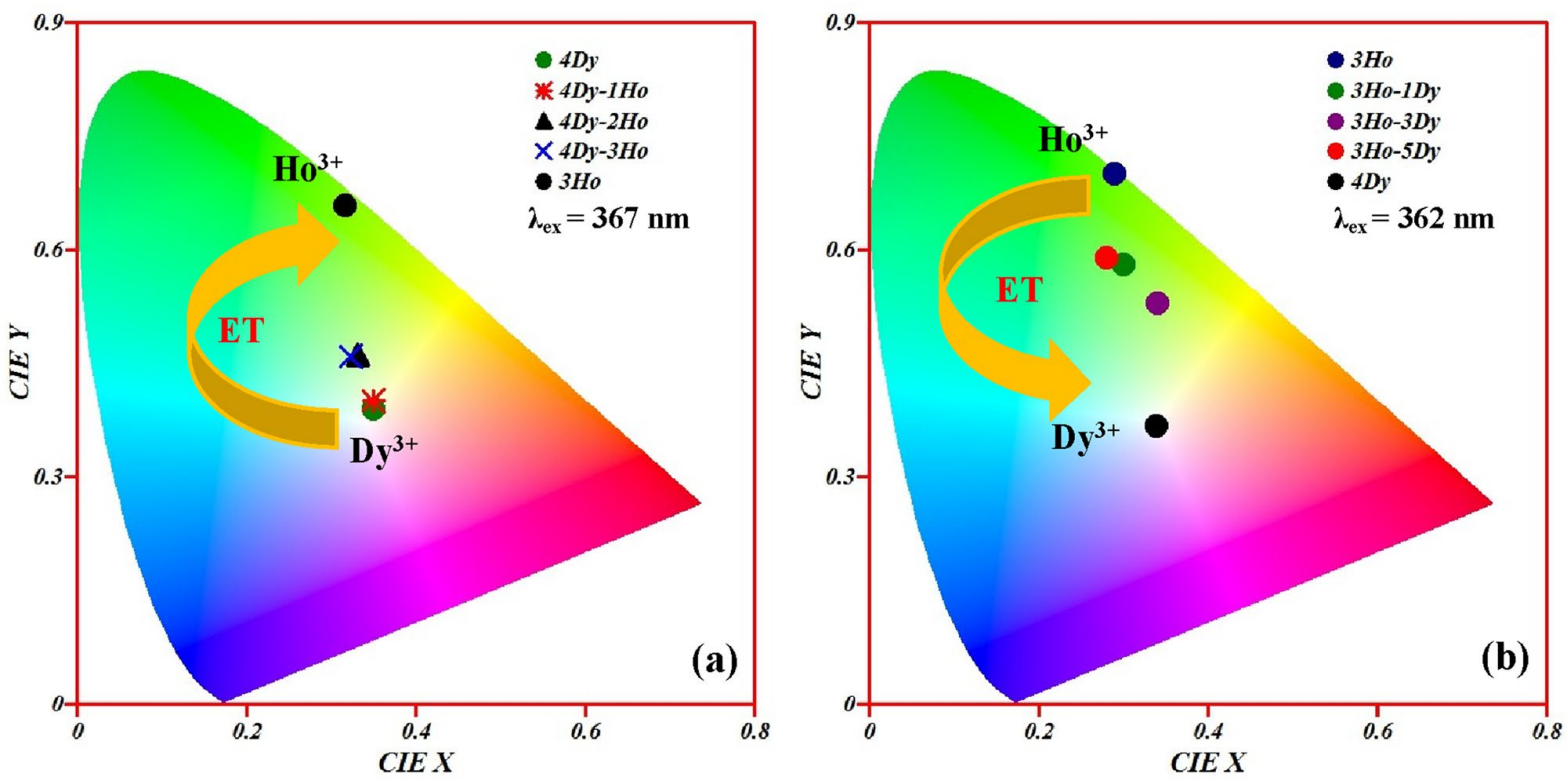
CIE diagram of (**a**) CaTiO_3_:4Dy^3+^/yHo^3+^ phosphors under 367 nm excitations and (**b**) CaTiO_3_:3Ho^3+^/xDy^3+^ phosphors under 362 nm excitation.

As we have seen that the Ho^3+^ doped CaTiO_3_ emits intense green, weak red and NIR emissions. Whereas, Dy^3+^ doped CaTiO_3_ sample emits blue and yellow emissions. So if both the rare earth ions are doped together in CaTiO_3_ host, it gives multicolor emission on excitation with 362/367 nm wavelengths. The CIE (commission Internationale de I’Eclairage) diagram is an excellent tool to verify the multicolor emitted from the doped/co-doped phosphors^2^. When only Dy^3+^ ions are present in the CaTiO_3,_ the CIE coordinates lie in whitish region on excitation with 367 nm. However, if yHo^3+^ co-doped in CaTiO_3_:4Dy^3+^ phosphor, the color coordinates vary from whitish to greenish yellow region. On the other hand, CaTiO_3_:3Ho^3+^ phosphor gives intense green emission. The color coordinates vary from deep green to greenish yellow in CaTiO_3_:3Ho^3+^/xDy^3+^ phosphors under the 362 nm excitation. Figure 13a,b shows the CIE diagram of CaTiO_3_:4Dy^3+^/yHo^3+^ and CaTiO_3_:3Ho^3+^/xDy^3+^ phosphors under 367 and 362 nm excitation wavelengths, respectively.

**Figure 14:**
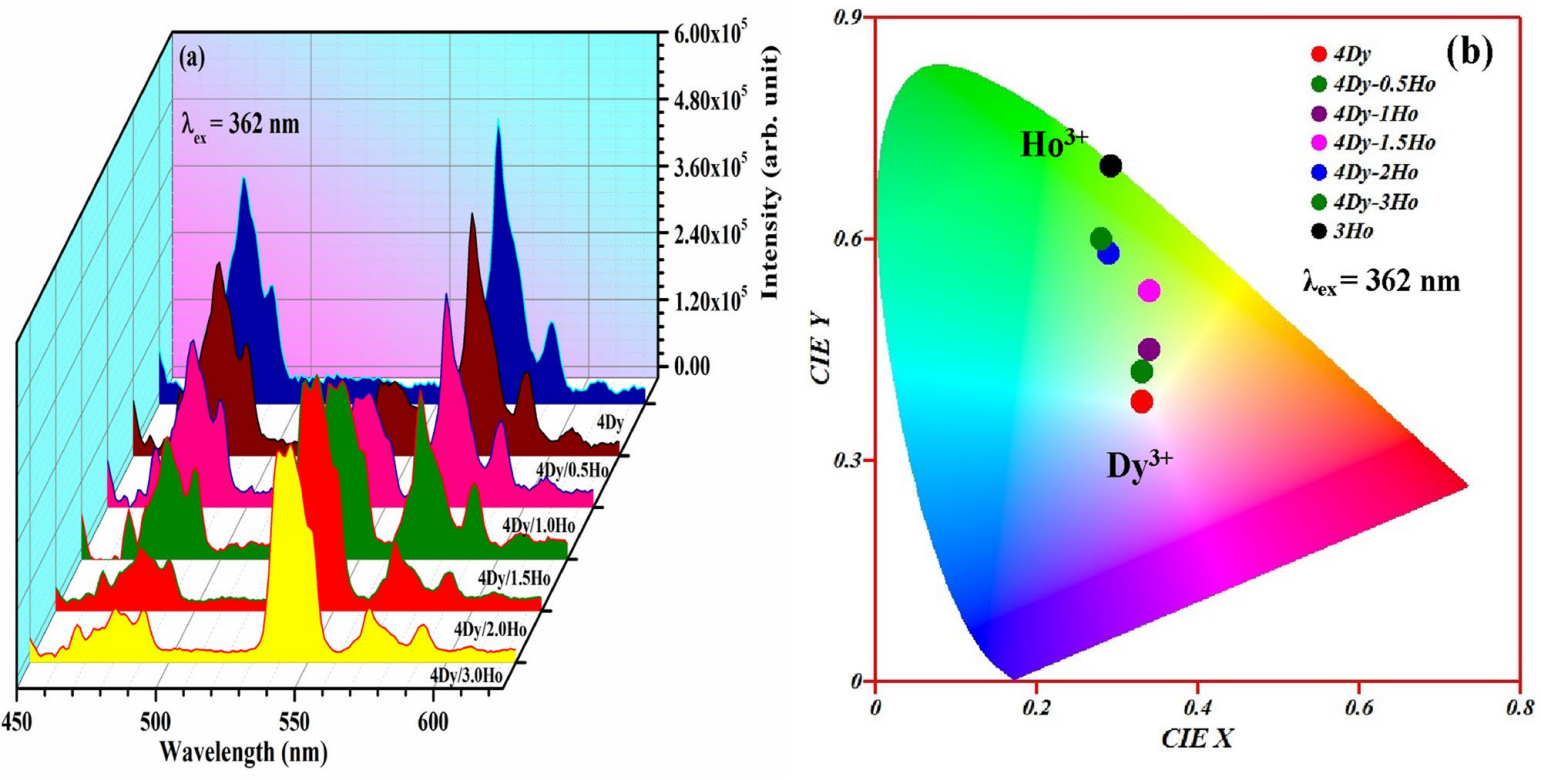
(**a**) Photoluminescence emission spectra and (**b**) CIE diagram of CaTiO_3_:4Dy^3+^/yHo^3+^ phosphors under 362 nm excitations.

It is interesting to observe that CaTiO_3_:4Dy^3+^/yHo^3+^ phosphors show excellent color tunability from whitish to green through greenish yellow under 362 nm excitation. Figure 14a,b shows the PL spectra and the CIE diagram for CaTiO_3_:4Dy^3+^/yHo^3+^ phosphors with λ_ex_ = 362 nm. The green emission intensity increased with increasing the Ho^3+^ ion concentration and the intensity of blue and yellow (Dy^3+^ ions) decreased. This occurs due to sharing of excitation energy in between Ho^3+^ and Dy^3+^ both ions. Thus for 0 mol% doping of Ho^3+^ in CaTiO_3_:4Dy^3+^/yHo^3+^ phosphors, the emission color lies in whitish region (due to pure Dy^3+^) with CIE coordinates (0.33, 0.38). When the Ho^3+^ concentrations in CaTiO_3_:4Dy^3+^/yHo^3+^ phosphors is increased from 0 to 3 mol %, CIE coordinates of the phosphors is changed from whitish (0.33, 0.38) to green (0.28, 0.60). This is also obvious from emission spectra in Fig. 14a.

The values of CIE coordinates for different concentrations of Ho^3+^ are given in Table 1. A similar thing is observed in CaTiO_3_:3Ho^3+^/xDy^3+^ phosphors and the color coordinates are given in Table 1. The variation in CIE coordinates clearly show the possibility of achieving multicolor tunability by adjusting the Dy^3+^/Ho^3+^ doping concentrations. Hence, Ho^3+^/Dy^3+^ co-doped CaTiO_3_ phosphors can be used in the field of single matrix perovskite tunable phosphor materials which are suitable for display devices, LED/WLEDs for solid state lighting applications.

**Table 1:** CIE coordinates and CCT values of Dy^3+^, Ho^3+^ and Dy^3+^/Ho^3+^ co-doped CaTiO_3_ phosphors under 362 and 367 nm excitation wavelengths.

Phosphors	Wavelengths (nm)	CIE Coordinates	CCT values	Color purity
4Dy^3+^	367	(0.35, 0.39)	4955	22.1
4Dy^3+^/1Ho^3+^	367	(0.35, 0.40)	4983	25.1
4Dy^3+^/2Ho^3+^	367	(0.33, 0.46)	5568	37.5
4Dy^3+^/3Ho^3+^	367	(0.32, 0.46)	5767	34.7
3Ho^3+^	367	(0.33, 0.66)	5549	98.2
3Ho^3+^	362	(0.29, 0.70)	6066	99.1
3Ho^3+^/1Dy^3+^	362	(0.30, 0.58)	6052	65.5
3Ho^3+^/3Dy^3+^	362	(0.34, 0.53)	5328	61.5
3Ho^3+^/5Dy^3+^	362	(0.28, 0.59)	6405	63.4
4Dy^3+^	362	(0.33, 0.38)	5596	13.2
4Dy^3+^/0.5Ho^3+^	362	(0.33, 0.42)	5588	25.4
4Dy^3+^/1.0Ho^3+^	362	(0.34, 0.45)	5333	37.4
4Dy^3+^/1.5Ho^3+^	362	(0.34, 0.53)	5328	61.6
4Dy^3+^/2.0Ho^3+^	362	(0.29, 0.58)	6238	62.9
4Dy^3+^/3.0Ho^3+^	362	(0.28, 0.60)	6382	66.3

The correlated color temperature (CCT) is used to evaluate the nature of emitted light from the phosphors i.e. whether it is warm, natural or cool white light. The CCT values are calculated using McCammys’ equation^2, 52^:

6 $${\text{T }} = \, - { 449 }\left( {{\text{n}}^{{3}} } \right) \, + { 3525 }\left( {{\text{n}}^{{2}} } \right) \, {-}{ 6823}.{3 }\left( {\text{n}} \right) \, + { 552}0.{33,}$$

where n = (*x* – 0.3320)/(*y* – 0.1858) and (*x*, *y*) refers the CIE coordinates. The calculated CCT values are given in Table 1. The CCT values lie between natural and cool white light range.

The color purity of the phosphor materials is another important parameter to recognize it as a good source of light for a particular color for solid state lighting applications. The color purity of the light source can be calculated by following relation^52^:

7 $$\mathrm{Color\, purity}=\frac{\sqrt{({x-{x}_{i})}^{2}+{(y-{y}_{i})}^{2}}}{\sqrt{({{x}_{d}-{x}_{i})}^{2}+ {({y}_{d}- {y}_{i})}^{2}}} \times 100\%,$$

where (*x*, *y*) are the CIE coordinates of the phosphor, (*x*_*d*_, *y*_*d*_) are the CIE coordinates of dominant wavelength and (*x*_*i*_, *y*_*i*_) are the CIE coordinates for standard white light source. The value of color purity is mentioned in the Table 1. The value of color purity varies from 13.2 to 66.3 for CaTiO_3_:4Dy^3+^/yHo^3+^ phosphors under 362 nm excitations.

### Lifetime measurements

**Figure 15:**
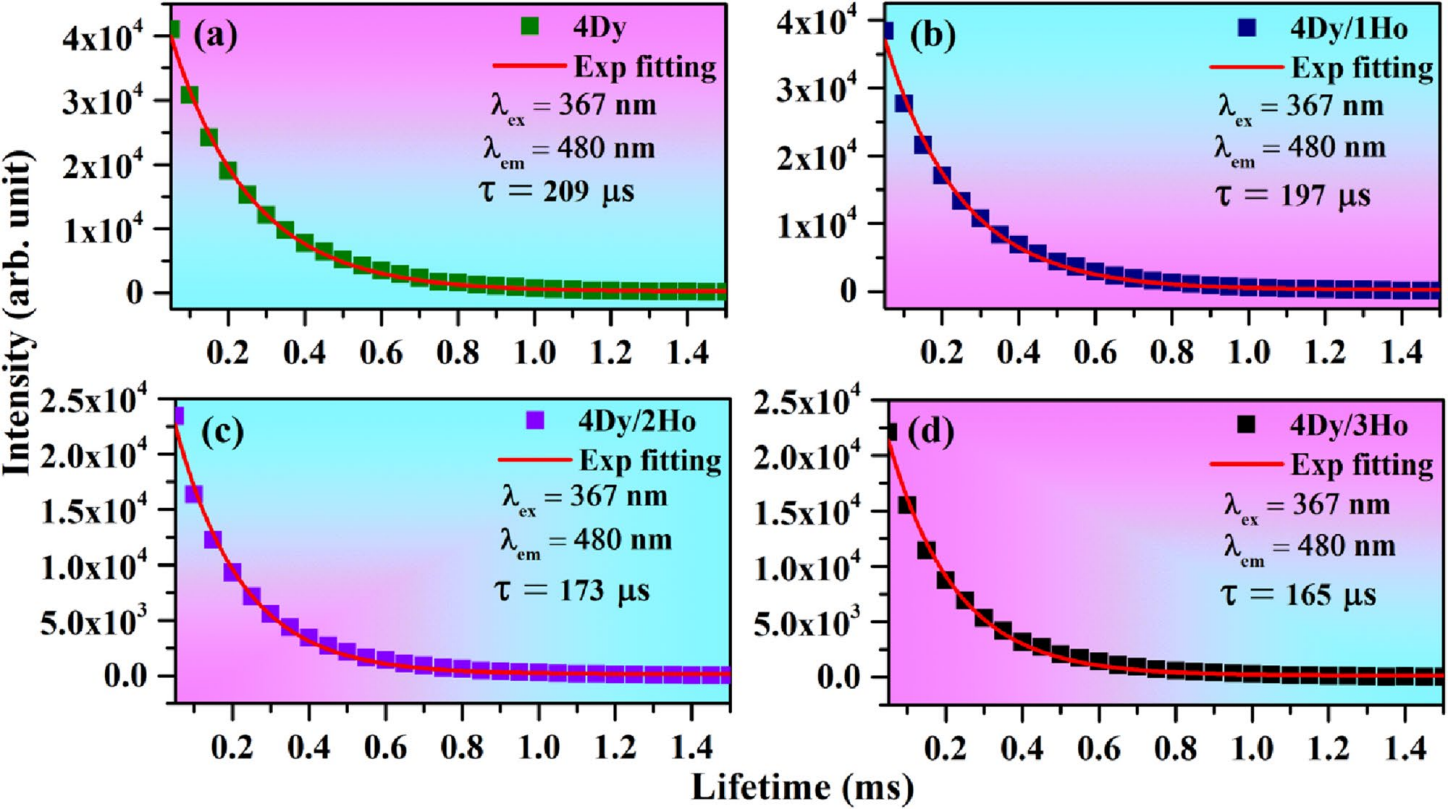
Lifetime of ^4^F_9/2_ level of Dy^3+^ in (**a**) 4Dy^3+^, (**b**) 4Dy^3+^/1Ho^3+^, (**c**) 4Dy^3+^/2Ho^3+^ and (**d**) 4Dy^3+^/3Ho^3+^ co-doped CaTiO_3_ with λ_ex_ = 367 nm and λ_em_ = 480 nm.

**Figure 16:**
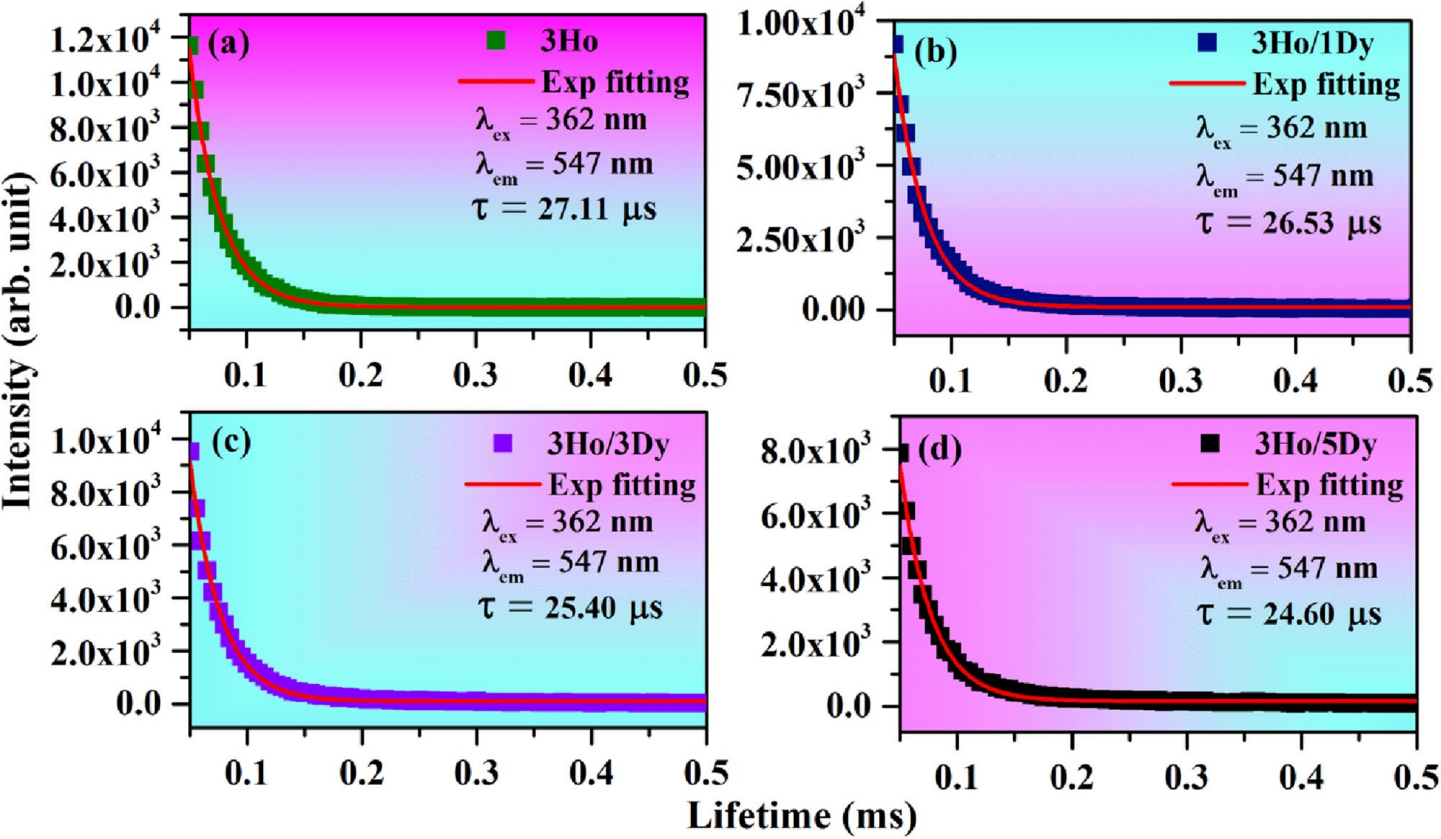
Lifetime of ^5^S_2_ level of Ho^3+^ in (**a**) 3Ho^3+^, (**b**) 3Ho^3+^/1Dy^3+^, (**c**) 3Ho^3+^/3Dy^3+^ and (**d**) 3Ho^3+^/5Dy^3+^ co-doped CaTiO_3_ with λ_ex_ = 362 nm and λ_em_ = 547 nm.

Lifetime study helps to understand the fundamental issues such as dopant location, surface defect etc. in the phosphor materials^53^. We have measured the lifetime of ^4^F_9/2_ level of Dy^3+^ in the absence and presence of Ho^3+^ ions with λ_ex_ = 367 nm and λ_em_ = 480 nm and the decay curves are shown in Fig. 15a–d. We have also measured the lifetime of ^5^S_2_ level of Ho^3+^ ion in the absence and presence of Dy^3+^ ion under λ_ex_ = 362 nm and λ_em_ = 547 nm and the decay curves are shown in Fig. 16a–d. It is found that all the decay curves fit well by single exponential formula^54^:

8 $${\text{I}}\left( {\text{t}} \right) \, = {\text{ I}}_{0} {\text{exp}}\left( { - {\text{t}}/\tau } \right),$$

where I_0_ and I(t) are the PL emission intensities at the time zero and at t seconds, respectively and ‘τ’ is the lifetime. It is interesting to note that even in co-doped cases the decay curves fit well with single exponential. If the decay curve needs multiexponential fitting, then probably there is defect also involved which must affect the lifetime of activator^55^. However, in our case even after the co-doping the decay curve fits well by single exponential. Therefore, we have concluded that there is no defect present in the materials. The values of lifetime are found to be 209, 197, 173 and 165 µs for 4Dy^3+^ doped and 4Dy^3+^/1Ho^3+^, 4Dy^3+^/2Ho^3+^, 4Dy^3+^/3Ho^3+^ co-doped CaTiO_3_ phosphors, respectively. However, the values of lifetime are found to be 27.11, 26.53, 25.40 and 24.60 µs for 3Ho^3+^ doped and 3Ho^3+^/1Dy^3+^, 3Ho^3+^/3Dy^3+^, 3Ho^3+^/5Dy^3+^ co-doped CaTiO_3_ phosphors, respectively. Thus, the lifetime for ^4^F_9/2_ level of Dy^3+^ ion decreased in presence of Ho^3+^ ions, due to energy transfer from Dy^3+^ to Ho^3+^ ions and the lifetime for ^5^S_2_ level of Ho^3+^ ion also decreased in presence of Dy^3+^ ions, due to energy transfer from Ho^3+^ to Dy^3+^ ions. Dwivedi et al. studied the downshifting and upconversion in Ho^3+^/Yb^3+^ co-doped GdNbO_4_ phosphor. They have also reported the lifetime of Ho^3+^ in microsecond (µs) under 449 nm excitation and 540 nm emission wavelengths^56^. Mahata et al. also reported the lifetime of Ho^3+^ ions in microsecond^57^.

### Temperature dependent photoluminescence and photoluminescence quantum yield (PLQY) measurements

**Figure 17:**
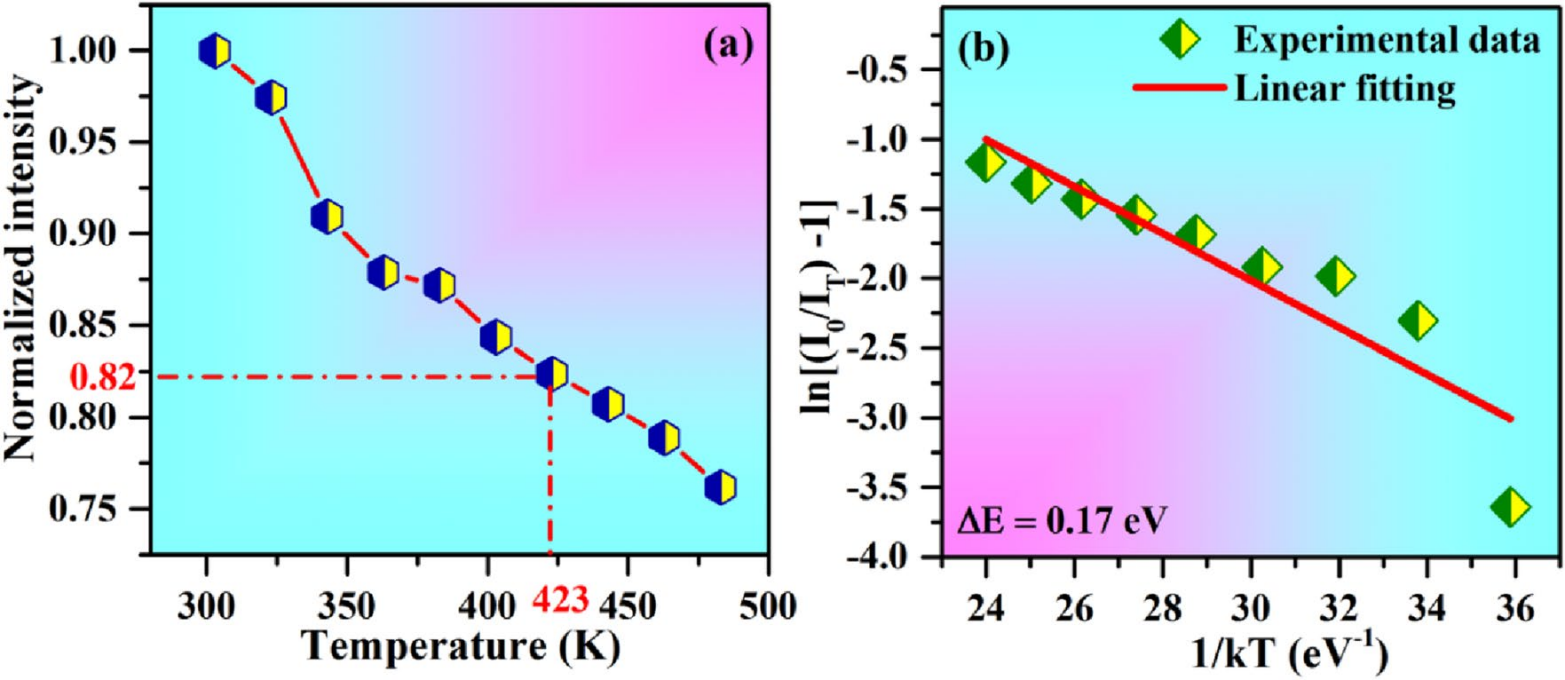
(**a**) An intensity plot as a function of temperature and (**b**) ln[(I_0_/I_T_)-1] vs 1/kT plot for CaTiO_3_:4Dy^3+^/2Ho^3+^ phosphor.

To know the thermal stability of the phosphor samples, we studied the temperature dependent photoluminescence emission spectra (TDPL) of CaTiO_3_:4Dy^3+^/2Ho^3+^ phosphor in the temperature range 303–483 K with 367 nm excitation. The thermal stability is very important for the applications of phosphor in industrial fields. The temperature dependent normalized PL emission intensity for CaTiO_3_:4Dy^3+^/2Ho^3+^ phosphor is depicted in Fig. 17a. As can be seen, the PL emission intensity decreased with the increase in temperature. This decrease in PL intensity is due to thermal quenching, which occurs due to non-radiative relaxation of phonons from higher excited states^4^. From Fig. 17a we found that the emission intensity at 423 K is 82% of 303 K for CaTiO_3_:4Dy^3+^/2Ho^3+^ phosphor. Hence the loss in emission intensity is only 18% at 423 K. This clearly shows that this phosphor material is highly stable. We have compared the thermal stability of our materials with the thermal stability of other Dy^3+^ doped/co-doped materials and it is given in the Table 2. The obtained value of thermal stability in present study has been higher than the other reported values [see Table 2]. Hence, CaTiO_3_:4Dy^3+^/2Ho^3+^ phosphor shows good thermal stability and can be used for LED applications.

**Table 2:** A comparison of the thermal stability for CaTiO_3_:4Dy^3+^/2Ho^3+^ phosphor with the thermal stability of other Dy^3+^ doped/co-doped phosphors.

Phosphor samples	Thermal stability (423 K)	References
CaTiO_3_:4 mol%Dy^3+^/2 mol%Ho^3+^	82%	Present study
BaLu_2_Si_3_O_10_: 0.04Dy^3+^, 0.1Eu^3+^	81.15%	^58^
Ba_3_Bi(PO_4_)_3_:8at.%Dy^3+^,0.8at.%Eu^3+^	70%	^59^
LiMgBO_3_:0.03Tm^3+^/0.05Dy^3+^/0.05Li^+^	61.5%	^60^
Sr_3_Gd(PO_4_)_3_:3%Dy^3+^,6% Eu^3+^	80%	^61^
InNbTiO_6_:0.06Dy^3+^	50%	^62^

The activation energy for thermal quenching process is calculated by Arrhenius equation^63,64^:

9 $$\frac{{I}_{T}}{{I}_{0}}= \frac{1}{1+{Aexp}^{\left(-\frac{\Delta E}{kT}\right)}},$$

where I_0_ is the initial emission intensity at 303 K, I_T_ is the emission intensity at temperature T K, ∆E is the activation energy, k is Boltzman constant (8.629 $\times$ 10^–5^ eV/K) and A is the constant frequency factor. The value of activation energy could be calculated from ln[(I_0_/I_T_) − 1] versus 1/kT plot and it is shown in Fig. 17b. The slope of the fitted line gives the value of activation energy. In the present case its value is found to be 0.17 eV for CaTiO_3_:4Dy^3+^/2Ho^3+^ phosphor. This clearly shows that this phosphor material is highly stable for various applications.

The photoluminescence quantum yield (PLQY) is an important parameter for the phosphor material to know its photoluminescence efficiency. The quantum yield is mathematically defined as the ratio of total number of photon emitted to the total number of photon absorbed under certain excitation^65,66^.

The absolute photoluminescence quantum yield of CaTiO_3_:4Dy^3+^, CaTiO_3_:4Dy^3+^/2Ho^3+^ and CaTiO_3_:3Ho^3+^ phosphors are monitored by using an integrating sphere with λ_ex_ = 367 and 452 nm, respectively. The value of PLQY is found to be 30%, 33% and 35% for CaTiO_3_:4Dy^3+^, CaTiO_3_:4Dy^3+^/2Ho^3+^ and CaTiO_3_:3Ho^3+^ phosphors, respectively. We have compared the PLQY of our materials with PLQY of other Dy^3+^ doped/co-doped phosphors and it is given in the Table 3**.** The obtained value of PLQY in present study has been higher than the PLQY of other Dy^3+^ doped/co-doped phosphors [see Table 3]. Hence, CaTiO_3_:4Dy^3+^, CaTiO_3_:4Dy^3+^/2Ho^3+^ and CaTiO_3_:3Ho^3+^ phosphors can be used for LED/WLED’s applications.

**Table 3:** A comparison of the PLQY values for CaTiO_3_:4Dy^3+^, CaTiO_3_:4Dy^3+^/2Ho^3+^ and CaTiO_3_:3Ho^3+^ phosphors with the PLQY of other Dy^3+^ doped/co-doped phosphors.

Phosphor samples	PLQY (%)	References
CaTiO_3_:3 mol%Ho^3+^	35	Present study
CaTiO_3_:4 mol%Dy^3+^	30	Present study
CaTiO_3_:4 mol%Dy^3+^/2 mol%Ho^3+^	33	Present study
SrAl_2_O_4_:1%Dy^3+^	9.52	^65^
InNbTiO_6_:0.06Dy^3+^	23.4	^62^
K_3_Y(PO_4_)_2_:2 mol%Dy^3+^	13	^66^
Na_3_La(PO_4_)_2_:2 mol%Dy^3+^	11	^66^
Na_5_Y_9_F_32_:1 mol%Dy^3+^/0.8 mol%Sm^3+^	15.9	^67^

## Conclusions

In this study, Dy^3+^, Ho^3+^ singly doped and Dy^3+^/Ho^3+^ co-doped CaTiO_3_ phosphor materials have been prepared by solid state reaction method at 1473 K. The prepared materials are characterized using structural and optical techniques. The phosphor samples show orthorhombic structure with Pnma(62) space group. The PL emission spectra of Dy^3+^ doped CaTiO_3_ gives intense blue and yellow emissions, while the Ho^3+^ doped CaTiO_3_ shows intense green emissions under UV LED wavelengths. A co-doping of the two ions together show an energy transfer from Dy^3+^ to Ho^3+^ as well as from Ho^3+^ to Dy^3+^ for CaTiO_3_:4Dy^3+^/yHo^3+^ and CaTiO_3_:3Ho^3+^/xDy^3+^ phosphors on λ_ex_ = 367 and 362 nm excitations, respectively. It is found that the energy transfers from Dy^3+^ to Ho^3+^ ion are due to quadruple-quadruple interaction whereas for Ho^3+^ to Dy^3+^ ion it is due to dipole–dipole. The energy transfer efficiency is found to be maximum 67.76% and 69.39% for CaTiO_3_:4Dy^3+^/3Ho^3+^ and CaTiO_3_:3Ho^3+^/5Dy^3+^ phosphors, respectively. The CIE color coordinates and the correlated color temperature (CCT) of the phosphors have been calculated, which show color tunability from whitish to green via greenish yellow color. The lifetime of ^4^F_9/2_ level of Dy^3+^ and ^5^S_2_ level of Ho^3+^ ions are decrease in presence of Ho^3+^ and Dy^3+^ ions, respectively. This is due to energy transfer from Dy^3+^ to Ho^3+^ ions and vice versa. Temperature dependent photoluminescence spectra measurement has been carried out to know the thermal stability of phosphor materials for various applications. The temperature dependent photoluminescence spectra of CaTiO_3_:4Dy^3+^/2Ho^3+^ phosphor shows high thermal stability (at 423 K is 82% of initial temperature 303 K) with activation energy 0.17 eV. We have also carried out the photoluminescence quantum yield (PLQY) measurements and it value in different cases are found to be 35% for CaTiO_3_:3Ho^3+^, 30% for CaTiO_3_:4Dy^3+^ and 33% for CaTiO_3_:4Dy^3+^/2Ho^3+^ phosphors. Hence, Dy^3+^ and Ho^3+^ singly doped and Dy^3+^/Ho^3+^ co-doped CaTiO_3_ phosphor materials can be used in field of single matrix perovskite color tunable phosphors which may be suitable for multicolor display devices, near UV chip excited LED/WLED’s for the solid state lighting applications and photodynamic therapy for cancer treatment.

## Experimental section

### Synthesis of materials

The phosphor samples were prepared by solid state reaction method. The starting chemicals were calcium carbonate (CaCO_3_, 99.9%), titanium dioxide (TiO_2_, 99.9%), dysprosium oxide (Dy_2_O_3_, 99.9%) and holmium oxide (Ho_2_O_3_, 99.9%). We prepared a series of samples of xDy^3+^ (x = 3.0, 4.0 & 5.0 mol %) and of yHo^3+^ (where y = 1.0, 3.0 & 5.0 mol %) doped CaTiO_3_ phosphors to get the particular concentration of Dy^3+^ and Ho^3+^ ions, respectively for optimum PL emission. At the next step mixed samples of CaTiO_3_:xDy^3+^:yHo^3+^ were synthesized to study the energy transfer and color tunability. For this we fixed the concentration of Dy^3+^ at 4 mol % (the concentration at which the emission intensity of Dy^3+^ was optimum) and varied the concentration of Ho^3+^ in CaTiO_3_:4Dy^3+^/yHo^3+^ (where y = 0.5, 1.0, 1.5, 2.0 & 3.0 mol %) phosphors and also by fixing the concentration of Ho^3+^ at 3 mol % and varying the concentration of Dy^3+^ in CaTiO_3_:3Ho^3+^/xDy^3+^ (where x = 1.0, 3.0 & 5.0 mol %) phosphors.

The pure chemicals were weighed carefully and mixed in an agate mortar using acetone as mixing medium for one hour. The samples were heated at 1473 K for 4 h in a programmable electrical furnace in air environment. The heated samples were cooled and crushed further to get fine powder. These powder samples were used for various characterizations.

### Instrumentations

The crystallization and phase detection of phosphors were made by XRD measurements using CuK_α_ radiation (λ = 0.15406 nm) with MiniFlex600 (Rigaku, Japan). The morphology of phosphors were studied using FE-SEM with the help of Zeiss, Evo 18 Research system. The energy dispersive X-ray spectroscopic (EDX) measurements were carried out to verify the elements present in the phosphor materials. The Fourier transform infrared (FTIR) measurements were carried out to know the vibrational groups present in phosphor samples using PerkinElmer I-Frontier system in the 400–3000 cm^−1^ region. The photoluminescence excitation and emission spectra of the materials were monitored by using Fluorolog-3 spectrophotometer with 450W Xenon lamp as source (Horiba Jobin Yvon). We have also measured the lifetime of ^4^F_9/2_ level of Dy^3+^ ion and ^5^S_2_ level of Ho^3+^ ion in absence and presence of Ho^3+^ and Dy^3+^ ion, respectively using 25W pulsed xenon lamp attached with the same unit (Fluorolog-3 spectrophotometer). The absolute PLQY measurement was done with the help of Horiba PTI QuantaMaster-400 fluorescence spectrometer equipped with an integrating sphere.

## Data Availability

The datasets used and/or analysed during the current study are available from the corresponding author on request.
